# High glucose levels promote glycolysis and cholesterol synthesis via ERRα and suppress the autophagy–lysosomal pathway in endometrial cancer

**DOI:** 10.1038/s41419-025-07499-y

**Published:** 2025-03-17

**Authors:** Xiaodan Mao, Lixiang Huang, Xianhua Liu, Xite Lin, Qibin Wu, Xinrui Wang, Yuan Ren, Jincheng Ma, Maotong Zhang, Yao Lin, Damian J. Ralser, Alexander Mustea, Gang Chen, Pengming Sun

**Affiliations:** 1 Laboratory of Gynecologic Oncology, Fujian Maternity and Child Health Hospital, College of Clinical Medicine for Obstetrics & Gynecology and Pediatrics, Fujian Medical University, Fuzhou, 350001 Fujian China; 2 Fujian Key Laboratory of Women and Children’s Critical Diseases Research, Fujian Maternity and Child Health Hospital (Fujian Women and Children’s Hospital), Fuzhou, 350001 Fujian China; 3 Fujian Clinical Research Center for Gynecological Oncology, Fujian Maternity and Child Health Hospital (Fujian Obstetrics and Gynecology Hospital), Fuzhou, 350001 Fujian China; 4 Pathology Department, Fujian Maternity and Child Health Hospital, College of Clinical Medicine for Obstetrics & Gynecology and Pediatrics, Fujian Medical University, Fuzhou, 350001 China; 5 Medical Research Center, Fujian Maternity and Child Health Hospital, College of Clinical Medicine for Obstetrics & Gynecology and Pediatrics, Fujian Medical University, Fuzhou, 350013 China; 6 Fujian-Macao Science and Technology Cooperation Base of Traditional Chinese Medicine-Oriented Chronic Disease Prevention and Treatment, Academy of Integrative Medicine, Fujian University of Traditional Chinese Medicine, Fuzhou, 350001 China; 7 Department of Gynecology and Gynecological Oncology, University Hospital Bonn, Venusberg-Campus 1, 53127 Bonn, Germany; 8 Department of Obstetrics and Gynecology, Tongji Hospital, Tongji Medical College, Huazhong University of Science and Technology, Wuhan, 430030 China

**Keywords:** Cancer metabolism, Metabolic syndrome

## Abstract

Endometrial cancer (EC) patients with Diabetes Mellitus (DM) always have a poor prognosis. Estrogen-related receptor α (ERRα) is known as the metabolic-related prognostic factor for EC. However, the mechanism linking glycolipid metabolism dysfunction mediated by ERRα to poor prognosis of EC with DM is still unclear. In vitro, high-glucose (HG) levels showed enhancement of ERRα expression, cell proliferation, and inhibition of the autophagic lysosomes and apoptosis by flow cytometry analysis, transmission electron microscopy, and CCK-8 assays. Mechanistically, lose-and-gain function assay, DNA sequencing, and CO-IP revealed HG increased ERRα expression to promote the transcription of *HK2* and *HMGCS1*, which were the key rate-limiting enzyme of glycolysis-cholesterol synthesis and their metabolites suppressed the autophagy–lysosomal pathway in an ERRα-dependent manner. Furthermore, CO-IP and molecular dynamics simulation uncovered the protein residues (ARG 769_HK2_ vs. ARG 313_HMGCS1_) of HK2 and HMGCS1 could bind to p62 to form stable protein complexes involved in the autophagy–lysosomal pathway. In EC tissue from patients with comorbid DM, ERRα was significantly higher expressed compared to EC tissue from patients without evidence for DM (*p* < 0.05). The 3D EC organoid model with HG stimulation showed that the cell viability of XCT790 + carboplatin treatment was similar to that of metformin+carboplatin treatment, while the obviously bigger volume of organoids was more visible in the metformin+carboplatin group, indicating the therapy of XCT790 + carboplatin had the better inhibition of EC organoids with the same carboplatin dose. Besides insights into the interaction of HG and the autophagy–lysosomal pathway via ERRα, our present study points out the potential benefit of targeting ERRα in patients with EC with dysregulation of glucose and cholesterol metabolism.

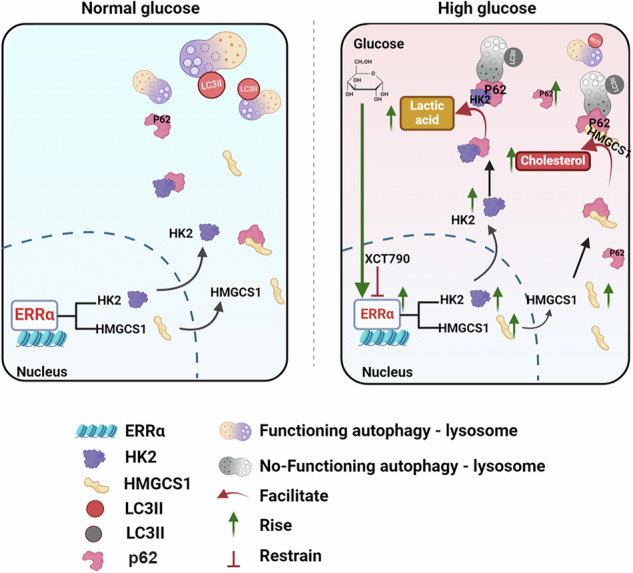

## Introduction

Endometrial cancer (EC) represents the sixth most prevalent malignancy among women worldwide, with increasing incidence [1, 2]. In China, 81,964 new cases of EC and 16,607 EC-related deaths were reported in 2020. Of note, despite significant improvements in EC treatment, EC-related morbidity and mortality have continued to increase recently [3]. Research has identified metabolic abnormalities like diabetes mellitus (DM) as risk factors for the development of EC. Women with DM exhibit a 72% increased risk of developing EC compared to women without DM [4]. Moreover, DM is strongly positively associated with EC-related death, with standardized mortality rates ranging from 1.08 to 2.40 [5, 6]. Further, there is broad scientific evidence that high blood glucose (HBG) levels promote lymphovascular interstitial infiltration (LVSI) and myometrial infiltration (MI) of endometrial cancer cells, thereby accelerating and significantly contributing to EC progression [7, 8].

Glucose is the main nutrient requirement for tumor cell growth. In this context, numerous previous studies have reported a connection between glucose metabolism and malignant tumor burden. In vitro, regulating glucose metabolism facilitates the control of tumor cell growth [9]. Further, glucose can function as a signaling molecule, directly activating oncogenes, thereby promoting tumorigenesis [10]. However, the precise underlying mechanism of how glucose levels impact tumor progression remains to be elucidated. Autophagy is a cellular process intimately related to the supply of energy [11]. Research has shown that inhibition of autophagy facilitates the growth and migration of pancreatic and breast cancer cells [12, 13]. In contrast, under nutrient deprivation conditions, glycolysis is inhibited, and autophagy is activated, thereby maintaining cell survival [14–16].

Estrogen-related receptor alpha (ERRα) is a nuclear receptor, and its expression is related to cellular energy metabolism [17, 18]. Previously, we demonstrated that ERRα is highly expressed in EC and closely correlated with myofibrillar infiltration, representing a marker for unfavorable clinical outcomes [19–21]. Considering that ERRα is a key moderator of cell metabolism, we aimed to investigate the influence of cell glucose- and cholesterol metabolism on ERRα expression and to evaluate the potential of ERRα to serve as a therapeutic target in EC. Therefore, in this study, cell culture and organoid models-based experiments were carried out.

## Methods

### Population samples

EC tissue specimens and blood samples were collected from a cohort of *n* = 661 patients admitted to Fujian Maternity and Child Health Hospital for surgical treatment of EC between 2017 and 2020. The inclusion criterion for the study was histopathologically confirmed as EC. Patients with preoperative treatment with chemotherapy, radiotherapy, immunotherapy, or the presence of concurrent malignancies other than EC were excluded (*n* = 119). Of the entire study cohort (*n* = 542), *n* = 80 patients had been diagnosed with DM. All patients that were enrolled in the study underwent a comprehensive medical history assessment, which included detailed inquiries regarding age, parity, menopausal status, and comorbidities such as hypertension, DM, and dyslipidemia. Each patient was assessed for blood pressure values, blood glucose (BG) levels, and serum lipid profile. Pathological characteristics and FIGO stage were documented based on the postoperative pathology report and intraoperative findings. The study protocol was approved by the Fujian Maternity and Child Health Hospital Ethics Commission (approval number 2021KLRD637).

### Cell line construction and culture

EC cell lines were treated with a medium supplemented with normal glucose (NG, 5.5 mM) or high glucose (HG, 25 mM). 19.5 mM Mnt (M813423 D-Mnt, AR, 98% d-mannitol) was added to the NG medium as an osmolality control. These media were added with 10% FBS (#10091148, Gibco, MT, USA) and 1% antibiotic-antifungal solution (#B120901, Basal Media, Shanghai, China). HEC-1A cells (KeyGEN BioTECH, Nanjing, China) were cultured in McCoy’s 5A Sugar-Free medium supplemented with glucose (#PM150718, Procell Life Science & Technology Co., Ltd., Wuhan, China). KLE cells (KeyGEN BioTECH, Nanjing, China) were incubated in a glucose-supplemented RPMI-1640 medium (#PM150122, Procell Life Science & Technology Co., Ltd., Wuhan, China). They were routinely cultured in a constant temperature incubator with 5% CO_2_ at 37 °C. HEC-1A and KLE cells were incubated with 5 µM and 10 µM simvastatin (indole dimethyl sulfoxide [DMSO]) (#Y190601, MP Biomedicals LLC, CA, USA) for 24 h, they were treated with 10 mM 2-deoxy-D-glucose (2-DG) (#HY-13966, MedChemExpress LLC, NJ, USA) for 24 h, or with 10 μM 3-bromopyruvic acid (3-BrPA) (#HY-19992, MedChemExpress LLC, NJ, USA) for 24 h.

### Lentiviral construction and cell modeling

For investigating the effects of ERRα up- and downregulation, respectively, ov-ERRα and si-ERRα lentiviruses were constructed and EC cell lines HEC-1A and KLE were infected with the viruses according to the indicated multiplicity of infection (MOI) for 24 h, after the medium was changed to complete medium. The fluorescence intensity of the viruses was observed by light microscopy after 72 h. The cell clusters were harvested for western blotting to detect alterations in the ERRα protein level resulting from transduction with the ov-ERRα and si-ERRα vectors. HEC-1A and KLE ERRα overexpressing cells are referred to as HEC-1A-ov-ERRα cells and KLE-ov-ERRα cells. Lentiviral vectors expressing siRNA targeting ERRα (labeled si-ERRα) were constructed and transduced into HEC-1A and KLE cells. si-ERRα‘s target sequence (GenBank accession number NM_004451.5) was 5-GAG CGA GAG TAT GTT CTA-3.

### CCK8 assay

After digestion and resuspension of adherent cells, the cell suspension was seeded into 96-well plates at a volume of 100 µl per well. Different glucose concentrations were incubated in 3 wells per group, along with a blank group and a control group. Then, 10 μl of CCK8 solution (#K1018, APExBIO, Huston, USA) was added to each well for the reaction, and the absorbance (OD) at 450 nm was measured using an enzyme marker.

### Transmission electron microscopy (TEM)

Centrifugation was utilized to collect cell precipitates, which were then fixed in an electron microscopy fixation solution (#G1102, Servicebio, Wuhan, China) at 4 °C for 2–4 h. Following fixation, the samples were preembedded in agar, dehydrated at room temperature, permeabilized in an embedded plate at 37 °C overnight, polymerized, sliced into ultrathin sections, stained, and dried on filter paper. The ultrathin sections were placed on copper mesh grids and dried absolutely before being observed under a TEM for image acquisition.

### Cell cycle analysis

Cell pellets were collected and resuspended in ethanol and refrigerated overnight at −20 °C. The cell suspension was then centrifuged, and 100 μl RNaseA was added to the cells. After ensuring that the cells were completely suspended, the reaction was carried out in a water bath at 37 °C for 30 min. Subsequently, 100 µl of propidium iodide (PI) (50 µg/mL) (#550825, BD Biosciences, NJ, USA) was added and mixed well, and the red fluorescence was immediately evaluated at an excitation wavelength of 488 nm.

### Apoptosis assay

Cell clusters were harvested following a 48 h exposure to various concentrations of glucose. The cell suspension was prepared using 1× binding buffer and then transferred 100 µL of cell suspension to a flow-through tube. 5 µL fluorochrome of Annexin V (APC) and 7-aminoactinomycin D (7-AAD) (#550475, BD Biosciences, NJ, USA) was added and then incubated in the dark for 15 min at room temperature, respectively. Afterward, 400 µl of 1× binding buffer was added, and flow cytometry analysis was carried out within one hour.

### Western blot analysis

Cells were treated with different concentrations of glucose for 48 h. Cells were lysed for protein analyses. Membranes were incubated with antibodies specific for ERRα (1:500, #ab137489, Abcam, MA, USA), HK2 (1:1000, #66974-1-Ig, Proteintech, Wuhan, China), HK2, (#ab209847, Abcam, MA, USA), PKM2 (1:1000, #60268-1-Ig, Proteintech, Wuhan, China), HMGCS1 (1:1000, #17643-1-AP, Proteintech, Wuhan, China), HMGCR (1:1000, #A16875, ABclonal, Wuhan, China), p62 (1:1000, #ab207305, Abcam, MA, USA) and β-actin (1:1000, #YM8343, ImmunoWay, Suzhou, China); washed; and incubated with a goat anti-rabbit secondary antibody (1:10,000, #SA00001-2, Proteintech, Wuhan, China) or goat anti-mouse secondary antibody (1:10,000, #SA00001-1, Proteintech, Wuhan, China) for 2 h. The membranes were washed 30 min. The membranes were then incubated with a chemiluminescence detection reagent for 2 min, removed from the solution, shaken to remove excess liquid, sensitized, and developed with X-ray film in a dark room.

### Lactic acid assay

Cells were treated with different concentrations of glucose for 48 h according to the protocol provided in the Lactate Assay Kit (#ab65331, Abcam, MA, USA). The cell pellet was collected in ethanol, lysed by ultrasound, and centrifuged for 10 min to obtain a supernatant for analysis. Thaw all reagents to room temperature or incubate in a 25 °C water bath for 10 min, then add 170 µL of the mixture to the plate. Warm up the enzyme marker for at least 30 min and set the wavelength to 450 nm.

### Total cholesterol assay

Cells were exposed to varying concentrations of glucose for 48 h according to the protocol provided in the Cholesterol Assay Kit (#ab102515, Abcam, MA, USA). Cells were then collected and treated with ethanol, followed by sonication and centrifugation for 10 min. The supernatant obtained was analyzed. Reagents are thawed to room temperature and added to a 96-well plate for detection. The enzyme counter was warmed up for 30 min and the instrument wavelength was set to 510 nm and calculated according to the formula.

### Cleavage under targets and tagmentation (CUT &Tag)

Based on our previous data, the endometrial cancer cell’s DNA sequence pulled down by ERRα was downloaded from https://bigd.big.ac.cn/gsa-human/browse/HRA007048. ChIPseeker was used to retrieve the nearest genes around the peak and annotate the genomic region of the peak. ChIPseeker can confirm peak-related genes[19].

### Coimmunoprecipitation (Co-IP)

Lysates were extracted and subsequently incubated with antibodies specific for HK2, HMGCS1, and P62. Agarose beads were combined to bind the antigen-antibody complexes, the bead-antigen-antibody complexes were precipitated by centrifugation. The complexes were denatured to allow their dissociation prior to analysis by western blotting.

### Molecular dynamics simulation

Production simulations were performed on the Amber 24 (San Francisco, CA, USA) [22]. ff12SB force field has been employed as the force field parameters of computers [23]. The TIP3P model has been employed for water, and the counterion liquids have been considered to neutralize the system. Molecular dynamics simulations have been performed in the isothermal−isobaric (NPT) ensemble. Once the system’s energy is minimized, it is heated from 0 K to 310.15 K (37 °C) within 500 ps. Constraints were imposed on the ensemble of collective coordinates, followed by a system equilibrium at 310.15 K. Finally, a 300 ns molecular simulation was carried out in an isothermal-isobaric ensemble with periodic boundary conditions. Hydrogen atoms were added assuming standard bond lengths and were constrained to their equilibrium position with the SHAKE. All MD simulations have been performed with AmberTools 23 [22, 24].

### Immunofluorescence (IF) staining

Cells were plated in a 24-well plate. After wall attachment, cells were incubated with various concentrations of glucose. Following 48 h, the cell culture medium was discarded, and the cells were fixed with 4% paraformaldehyde for 20 min at room temperature and permeabilized with 0.1% Triton X-100 for 20 min. The primary antibody was wet incubated overnight. The next day, the FITC-conjugated secondary antibody was incubated at 37 °C for 60 min and then with DAPI for 15 min in the dark. Sections were photographed by confocal microscopy.

### Organoid generation

Tumor tissues were cut into 1–3 mm cubic pieces, transferred into centrifuge tubes, and treated with tissue digestion solution. Tubes were then placed in a thermostatic shaker (37 °C, 100 rpm) for tissue digestion. When digestion was terminated, an equal volume of organoid culture medium containing 5% FBS was added. The cell sediment was mixed thoroughly with the matrix gel and inoculated into pre-warmed 24-well cell culture plates. The 24-well plates are then placed in a CO_2_ incubator and kept at 37 °C and 5% CO_2_ for 30 min. Subsequent medium changes were conducted every 3 days, with vigilant monitoring of organoid growth to ensure timely passaging.

### Statistical methods

Statistical analysis was conducted utilizing SPSS 23.0 software (IBM SPSS, Armonk, NY, USA) and GraphPad Prism 8 for data visualization. ImageJ was used for plotting data. Each experiment was carried out in triplicate. Sample size calculations were performed using the PASS software. Based on a pre-specified effect size of Cohen’s *w* = 0.3, we calculated the minimum sample size required to ensure a statistical efficacy of 80% (1 − *β* = 0.8), with a significance level set at 0.05. Continuous variables were reported as means ± standard deviations (SD) or medians with interquartile range (IQR). Categorical variables were presented as percentages. The chi-squared test and Fisher exact test were used for the comparison of categorical variables. A *t*-test was used for the analysis of cell growth, an analysis of variance (ANOVA) was used for the analysis of western blotting and flow cytometry data, and paired *t*-tests were used for the analysis of protein expression as determined by immunohistochemistry (IHC). All statistical analyses use a significance level of *p* < 0.05.

## Results

### EC patients with DM are more prone to myometrial infiltration (MI)

Clinicopathological data of *n* = 542 EC patients were collected. The study cohort was divided into two groups: (i) the presence of DM and (ii) no evidence for DM and the association of blood glucose levels and other clinical characteristics were analyzed. Among the *n* = 542 patients in our cohort, *n* = 80 (14.76%) were diagnosed with DM. Diagnosis of DM was associated with menopause status, hypertension, EC with MI, higher BMI, serum total cholesterol (TC), and serum triglyceride (TG) values (all (*p* < 0.05) (Table 1). Regarding MI, after adjusting for age, menopausal status, and BMI, FIGO stage (OR: 6.09, 95% CI: 3.69–10.06), DM (OR: 2.28, 95% CI: 1.36–3.82) were found as the independent risk factor. Besides, TC (OR: 1.49, 95% CI: 0.98–2.26) was a potential risk factor for MI (Table 2).

**Table 1:** Clinicopathologic feature of endometrial cancer with or without diabetes mellitus.

Characteristics	EC without DM (*n* = 462)	EC with DM (*n* = 80)	*p* Value
Age at diagnosis, median (IQR)	53 (49, 58)	59 (56, 62.25)	**<0.001**
Menopausal status, *n* (%)			**<0.001**
No	246 (53.2%)	65 (81.2%)	
Yes	216 (46.8%)	15 (18.8%)	
BMI, median (IQR)	24.07 (21.965, 26.497)	25.242 (22.885, 27.033)	**0.031**
Hypertension			**<0.001**
No	344 (74%)	28 (35%)	
Yes	118 (26%)	52 (65%)	
Total Cholesterol, *n* (%)			**<0.001**
≥5.2 (mmol/L)	314 (68%)	37 (46.2%)	
<5.2 (mmol/L)	148 (32%)	43 (53.8%)	
Triglyceride, *n* (%)			**0.006**
<1.7 (mmol/L)	320 (69%)	43 (54%)	
≥1.7 (mmol/L)	142 (31%)	37 (46%)	
CA125 (U/ml), median (IQR)	20.8 (14, 40.9)	23.2 (12.2, 38.3)	0.855
ER expression, *n* (%)			0.222
Low	197 (43.3%)	28 (35.9%)	
High	258 (56.7%)	50 (64.1%)	
PR expression, *n* (%)			0.404
Low	198 (43.5%)	30 (38.5%)	
High	257 (56.5%)	48 (61.5%)	
FIGO stage, *n* (%)			0.188
I–II	71 (15.4%)	17 (21.2%)	
III–IV	391 (84.6%)	63 (78.8%)	
CSI, *n* (%)			0.579
No	376 (81.4%)	63 (78.8%)	
Yes	86 (18.6%)	17 (21.2%)	
MI, *n* (%)			**<0.001**
No	353 (76.4%)	45 (56.2%)	
Yes	109 (23.6%)	35 (43.8%)	
LVSI, *n* (%)			0.821
No	394 (85.3%)	69 (86.2%)	
Yes	68 (14.7%)	11 (13.8%)	
LNM, *n* (%)			0.795
No	420 (90.9%)	72 (90%)	
Yes	42 (9.1%)	8 (10%)	

The bold values in the tables mean that the *p* value ＜0.05, which present the significant difference between the groups.

*EC* endometrial cancer, *DM* diabetes mellitus, *BMI* body mass index, *CA125* cancer antigen 125, *ER* estrogen receptor, *PR* progesterone receptor, *FIGO* international Federation of Gynecology and obstetrics, *CSI* cervical stromal involvement, *MI* myometrial infiltration, *LVSI* lymphovascular–interstitial infiltration, *LNM* lymph node metastasis, *p* < 0.05 is considered significantly different with bold value .

**Table 2:** Risk factors for myometrial infiltration in the patients with and without diabetes mellitus.

Characteristic	Univariable			Multivariable		
	OR	95% CI	*p*-Value	OR	95% CI	*p*-Value
Age at diagnosis	1.04	1.01, 1.06	0.008	1.02	0.99, 1.06	0.247
*Menopausal status*						
Premenopause	–	–		–	–	
Postmenopause	1.77	1.19, 2.65	0.005	1.44	0.84, 2.48	0.187
BMI	0.97	0.92, 1.02	0.282	0.96	0.91, 1.02	0.201
*History of diabetes*						
Absent	–	–				
Present	2.52	1.54, 4.12	<0.001	2.28	1.36, 3.82	**0.002**
*History of hypertension*						
Absent	–	–				
Present	1.4	0.94, 2.09	0.101			
*FBG (mmol/L)*						
<6.1	–	–				
≥6.1	1.44	0.90, 2.30	0.125			
*TG (mmol/L)*						
<1.7	–	–				
≥1.7	1.21	0.81, 1.80	0.359			
*TC (mmol/L)*						
<5.2	–	–		–	–	
≥5.2	1.45	0.96, 2.20	0.076	1.49	0.98, 2.26	**0.061**
*FIGO stage*						
I–II	–	–				
III–IV	5.35	3.31, 8.65	<0.001	6.09	3.69, 10.06	**<0.001**

*EC* endometrial cancer, *DM* diabetes mellitus, *BMI* body mass index, *FBG* fast blood glucose, *TG* triglyceride, *TC* Cholesterol, *FIGO* international Federation of Gynecology and obstetrics, Adjust for age, Menopausal status and BMI. OR (95% CI): odds ratio (95% confidence interval). *p* < 0.05 is considered significantly different with bold value.

### Glucose promotes EC cell proliferation and inhibits autophagic lysosome (ASS) formation and apoptosis

**Fig. 1:**
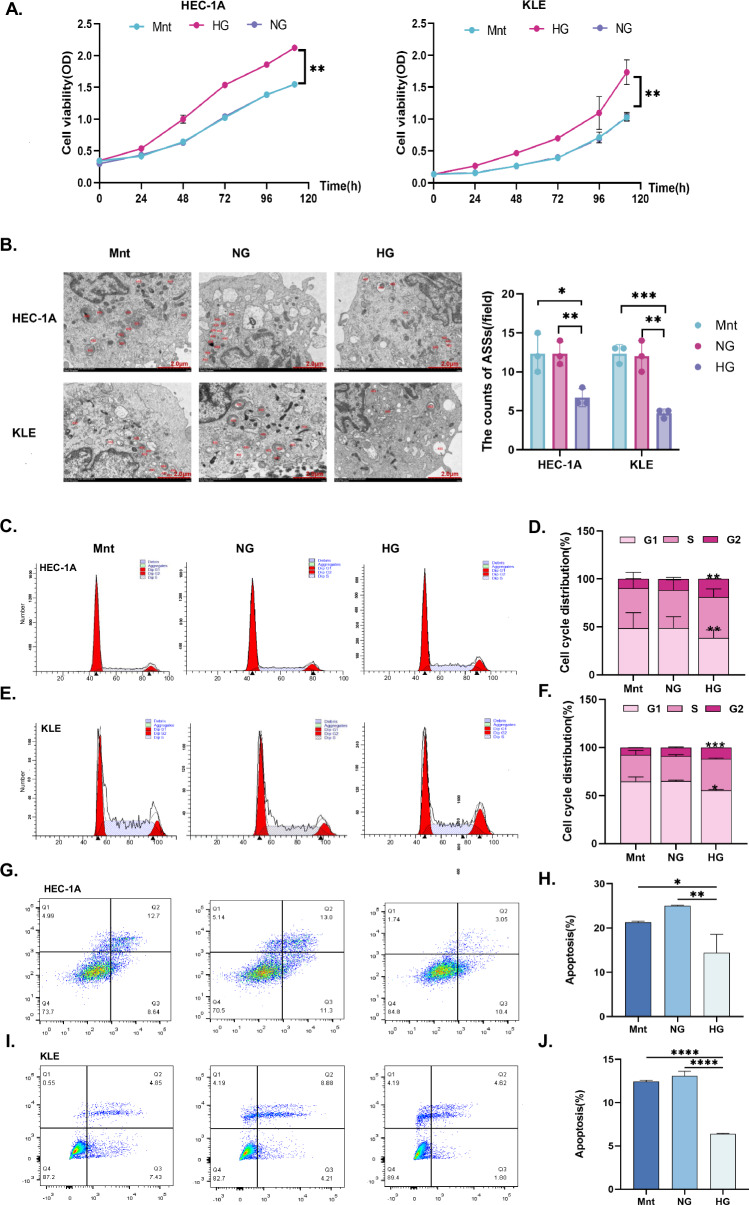
High concentrations of glucose promote EC cell proliferation and inhibit autophagy and apoptosis. **A** HEC-1A and KLE EC cells were treated with Mnt, NG (5.5 mM) medium, or HG (25 mM) medium for specific times (24 h, 48 h, 72 h, 96 h, 120 h), and cell viability was determined by a CCK8 assay. **B** Transmission electron microscopy (TEM) was performed to observe the effect of Mnt, NG, and HG on the number of ASSs in HEC-1A and KLE EC cells for 48 h. The red marks indicate ASSs (Magnification: 8000X). **C**–**F** The effects on the cell cycle in HEC-1A and KLE EC cells treated with Mnt, NG, or HG for 48 h were evaluated by flow cytometry, respectively. **G**–**F** The effects on the apoptosis in HEC-1A and KLE EC cells treated with Mnt, NG, or HG for 48 h were evaluated by flow cytometry, respectively. *n* = three independent experiments, * *p* < 0.05, ** *p* < 0.01, *** *p* < 0.001. EC endometrial cancer, Mnt mannitol, NG normal glucose, HG high glucose, ASS autophagic lysosome.

To simulate different glucose microenvironments, the normal glucose (NG, 5.5 mM glucose) and high glucose (HG, 25 mM glucose) groups were established. To exclude the effects of a difference in osmotic pressure between the HG and NG groups, we added 19.5 mM mannitol (Mnt) to the NG group to establish the Mnt group [7, 25, 26]. The proliferation rates of the EC cell lines HEC-1A and KLE in the HG groups were higher than that in the NG groups and the Mnt groups (*p* < 0.01, Fig. 1A); however, they did not differ significantly between the NG groups and the Mnt groups. Thus, the effect of osmolality could be neglected. ASS is a marker of advanced autophagy [27]. We determined the number of ASSs by using TEM. The number of ASSs in each HG group was significantly lower than in the corresponding Mnt and NG groups (*p* < 0.05, Fig. 1B). In addition, the G2 phase was significantly longer, and the G1 phase was shorter in the HG groups than that in the Mnt and NG groups (*p* < 0.05, Fig. 1C–F). Moreover, early and late apoptosis rates in the HG groups were lower than those in the other groups (*p* < 0.05, Fig. 1G–J). These findings confirmed that the HG microenvironment could promote EC cell proliferation, inhibit apoptosis, and reduce autophagy.

### Glucose inhibits the ASS pathway in an ERRα-dependent manner

**Fig. 2:**
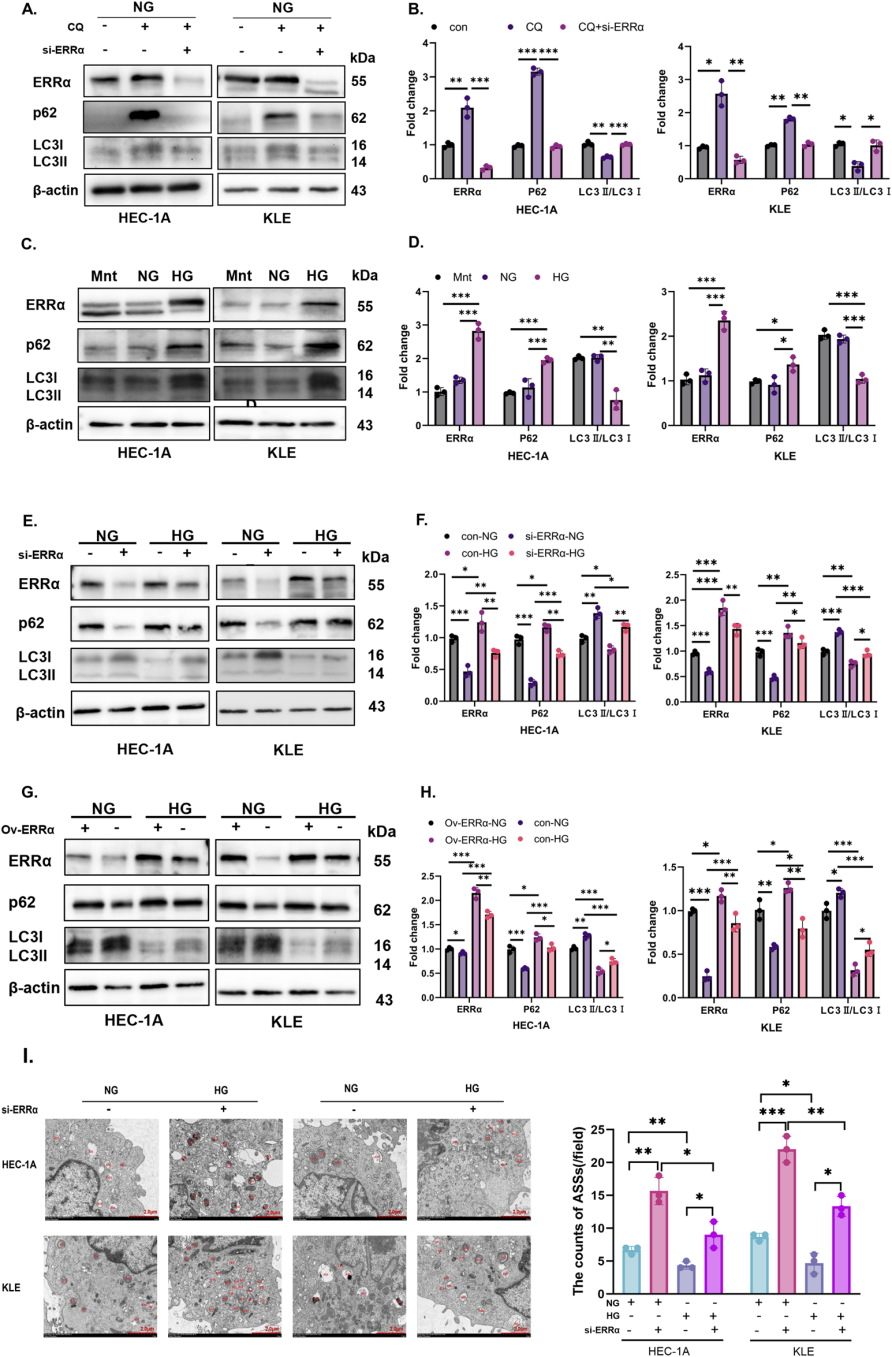
High-glucose induced ERRα increase to inhibited autophagy pathway. **A**, **B** HEC-1A and KLE were treated with chloroquine before and after ERRα down-regulating under NG condition, the protein levels of ERRα, p62 and LC3II/LC3I were measured by western blotting, with β-actin used as a loading control for statistical analysis (analysis of variance). **C**, **D** HEC-1A and KLE were treated with Mnt, NG, or HG for 48 h, and the protein levels of ERRα, p62, and LC3II/LC3I were measured by western blotting, with β-actin used as a loading control for statistical analysis (analysis of variance). **E**, **F** HEC-1A and KLE transduced with si-ERRα were treated with NG or HG for 48 h, and the protein levels of ERRα, p62, and LC3II/LC3I were measured by western blotting, with β-actin used as a loading control for statistical analysis (analysis of variance). **G**, **H** HEC-1A and KLE EC cells with ov-ERRα were subjected to NG or HG conditions for 48 h, and the protein levels of ERRα, p62, and LC3 were measured by western blotting, with β-actin used as a loading control for statistical analysis (analysis of variance). **I** Changes in the number of ASSs after modulation of ERRα expression were observed by TEM (Magnification: 8000×). *n* = three independent experiments, *P < 0.05, **P < 0.01 or ***P < 0.001. Mnt mannitol, NG normal glucose, HG high glucose, EC endometrial cancer, ASS autophagic lysosome, si-ERRα ERRα is knockdown, ov-ERRα ERRα is over-expressed.

To investigate the effect of an HG microenvironment on ERRα expression and autophagy, we measured the protein expression levels of ERRα, p62, and LC3II/LC3I in the Mnt, NG, and HG groups. Firstly, it has demonstrated that when chloroquine, an autophagy inhibitor, was added, autophagy in EC cells can be significantly inhibited, and the autophagy inhibition can be significantly weakened by downregulation of ERRα with the NG culturing (*p* < 0.05, Fig. 2A, B). Surprisingly, the expression of ERRα doubled, p62 expression increased, and the LC3II/LC3I levels were lower in the HG group compared to Mnt and NG groups. However, Mnt had no effect on ERRα, p62, or LC3II/LC3I protein levels, which suggested that an HG microenvironment can induce the ERRα expression and affect autophagy (*p* < 0.05, Fig. 2C, D). Moreover, there was a correlation between ERRα and autophagy in cells under HG stimulation. We found that downregulation of ERRα results in decreased expression of p62 and increased expression of LC3II/LC3I protein levels in EC cells treated with HG (*p* < 0.05, Fig. 2E, F). This pattern was vice versa when ERRα was overexpressed (Fig. 2G, H). In addition, TEM was used to compare the number of ASSs between the NG and HG groups following ERRα knockdown. The number of ASSs was highest in the NG group of ERRα-knockdown (si-ERRα) cells and lowest in the HG group of cells without ERRα knockdown (Fig. 2I). These data suggest that ERRα expression is a sensitive surrogate for extracellular glucose levels and that glucose inhibits autophagy in a manner that is partially dependent on ERRα expression.

### ERRα mediated glucose stimulation accelerates tumor glycolysis and cholesterol synthesis

**Fig. 3:**
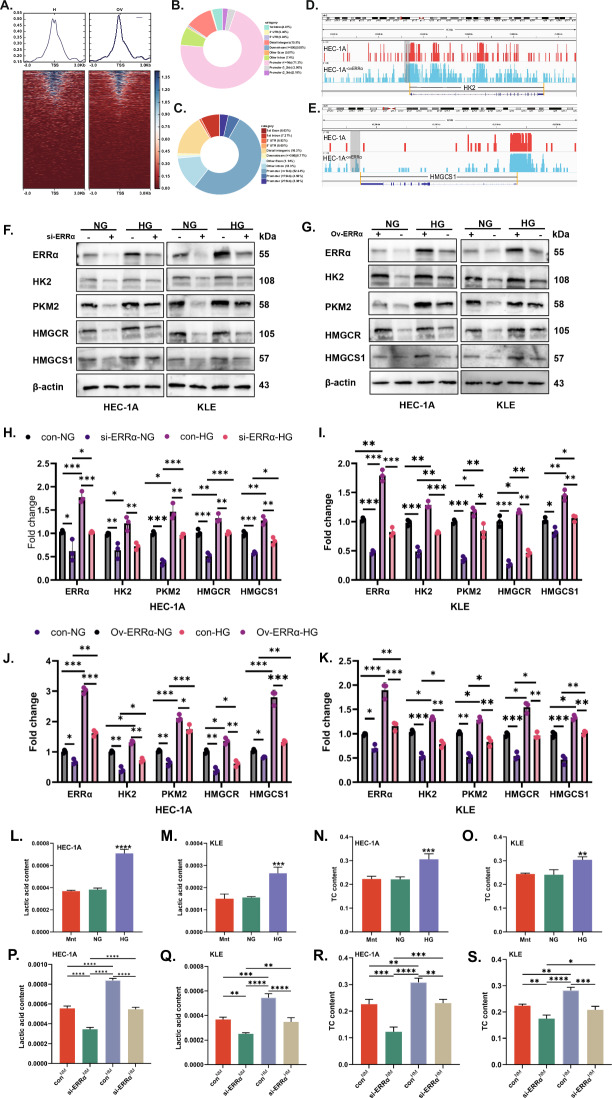
After stimulation by high glucose, the role of ERRα in regulating glucose and cholesterol metabolism. **A** Heatmap of the average read density and chromatin occupancy of the transcription start site (TSS) of ERRα in two independent samples of HEC-1A cells with control or upregulated ERRα. **B**, **C** Distribution of CUT&Tag peaks of ERRα in the genomic region from the second repetition of control samples. **D**, **E** Distribution of ERRα sites relative to HK2 and HMGCS1 promoters based on the CUT&Tag analysis. **F** HEC-1A and KLE EC cells transduced with si-ERRα were treated with NG or HG for 48 h. The expression of ERRα, HK2, PKM2, HMGCR, and HMGCS1 was measured by western blotting (WB), with β-actin used as a loading control. **G** HEC-1A and KLE EC cells with ov-ERRα were subjected to NG or HG for 48 h. The expression of ERRα, HK2, PKM2, HMGCR, and HMGCS1 was measured by western blotting, with β-actin used as a loading control. **H**, **I** Analysis of the data in panel F comparing the differences in protein expression among the si-ERRα-NG, con-HG, and si-ERRα-HG groups using the con-NG group as the reference baseline. **J**, **K** Analysis of the data in panel G comparing the differences in protein expression among the con-NG, ov-ERRα-HG, and con-HG groups using the ov-ERRα-NG group as the reference baseline. **L**–**O** HEC-1A and KLE EC cells were treated with Mnt, NG, or HG for 48 h, after which the lactate or TC concentrations were measured with three replications, respectively. **P**, **S** HEC-1A and KLE EC cells with or without ERRα knockdown were treated with NG or HG for 48 h, after which the lactate or TC concentration was measured with three replications, respectively. *P < 0.05, **P < 0.01 or ***P < 0.001. NG normal glucose, HG high glucose, EC endometrial cancer, TC total cholesterol.

Disorders of glucose metabolism are associated with dyslipidemia [28, 29]. ERRα not only acts as a hub of energy metabolism but also as a transcription factor [30]. ERRα-related CUT & Tag analysis had been performed to identify the transcriptional relationship between ERRα and HK2 or HMGCS1. The average read density and chromatin occupancy heatmap of the transcription start site are shown in Fig. 3A–C. There was the distribution of peaks in genomic regions in the sequencing results of the over-regulated ERRα groups and its control, which suggested the distribution of ERRα loci with respect to HK2 and HMGCS1 promoters (Fig. 3D, E). Moreover, increased expression of HK2, PKM2, HMGCR, and HMGCS1 were observed in response to HG stimulation. The effects of ERRα expression on HK2, PKM2, HMGCR, and HMGCS1 protein expression following HG stimulating were determined by loss and gain of ERRα function. It was demonstrated that the expression of HK2, PKM2, HMGCR, and HMGCS1 increased with ERRα expression increasing, which showed a positive correlation with ERRα expression under normal glucose. Under high glucose conditions, downregulation of ERRα can significantly inhibit the expression of HK2, PKM2, HMGCR, and HMGCS1 (all *p* < 0.01, Fig. 3F, H, I). While upregulation of ERRα can obviously increase the HK2, PKM2, HMGCR, and HMGCS1 in HG groups compared with NG groups (all *p* < 0.01, Fig. 3G, J, K). These indicated that high glucose-stimulated the expression of key enzymes involved in glycolysis (HK2 & PKM2) and cholesterol synthesis (HMGCR & HMGCS1) in EC cells in an ERRα-dependent manner. However, data on the relationships between metabolites and dysregulation of glycolysis in EC patients are sparse. Therefore, we used a complete medium supplemented with glucose at different concentrations to culture HEC-1A and KLE cells for 48 h, collected the cell clusters, then, measured the concentrations of lactate and TC in the EC cells. Interestingly, the levels of lactate (Fig. 3L, M) and TC (Fig. 3N, O) were increased in both cells with high glucose stimulation (all *p* < 0.01). Moreover, ERRα down-regulated decreased the levels of intracellular lactate (Fig. 3P, Q) and cholesterol (Fig. 3R, S) under normal glucose in both cells (all *p* < 0.01).

### ERRα-regulated key metabolic enzymes cooperate with p62 to restrain ASSs

**Fig. 4:**
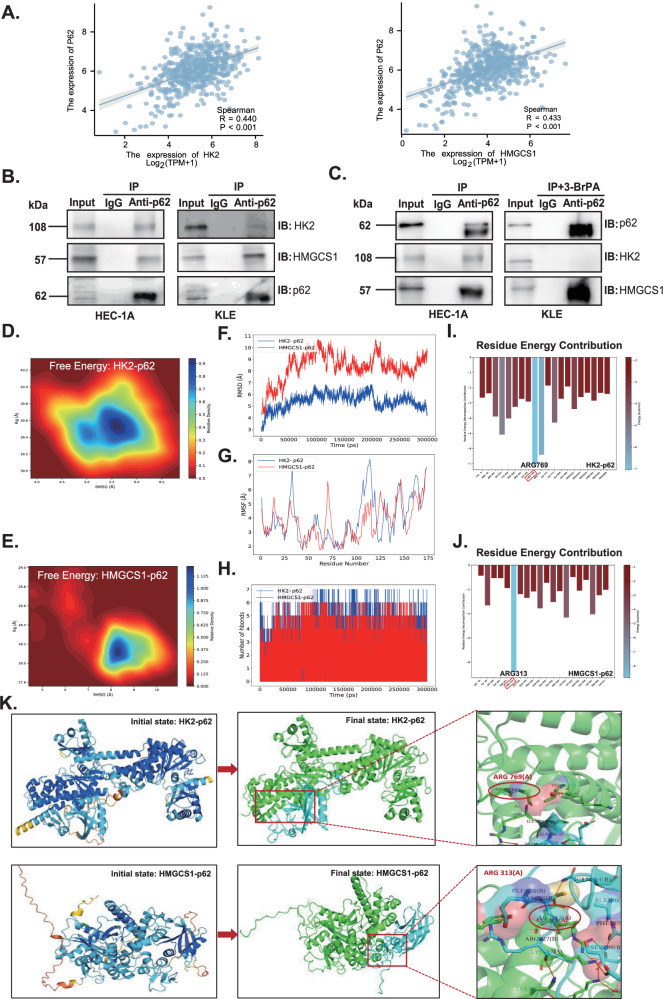
PPI between HK2 or HMGCS1 and p62 protein and their molecular dynamics simulation analysis. **A** The correlation between P62 and HK2, HMGCS1 expression was analyzed by TCGA database. **B** Co-IP was performed to identify the interaction between HK2 or HMGCS1 and p62 in HEC-1A and KLE cells. **C** Co-IP was performed to clarify the relationship between HK2 and HMGCS1 binding with p62 when added in 10 μM 3-BrPA in KLE cells. **D** The free energy analysis for the complex of HK2 and p62. **E** The free energy analysis for the complex of HMGCS1 and p62. **F** The RMSD analysis for HK2–p62 and HMGCS1-p62 complexes. **G** RMSF analysis for HK2–p62 and HMGCS1–p62 complexes. **H** Hydrogen bonds analyze for HK2–p62 and HMGCS1–p62 complexes. **I** The residue energy contribution analyze for HK2–p62 complex. **J** The residue energy contribution analyze for HMGCS1–p62 complex. **K** Visualization of the entire MDS process of the HK2–p62 and HMGCS1–p62 complexes, respectively. PPI protein–protein interaction, CO-IP coimmunoprecipitation, 3-BrPA 3-bromopyruvic acid (the inhibitor of HK2), MDS molecular dynamics simulation.

Bioinformatic analysis revealed positive correlations between HK2 and P62 expression and between HMGCS1 and P62 expression (*p* < 0.05, Fig. 4A). Moreover, protein interactions among HK2, HMGCS1, and p62 were verified in HEC-1A and KLE cells by CO-IP (Fig. 4B). Besides, there was a significant increase of HMGCS1 interacted with p62 when HK2 was inhibited by 10 μM 3-Bromopyruvic acid (3-BrPA), the inhibitor of HK2, in KLE cells for 24 h. It was indicated that HMGCS1 and HK2 affected the ability of each other’s binding to p62, probably (Fig. 4C). To uncover the interactions between proteins and their dynamics, molecular dynamic simulations (MDS) were performed. The protein-protein interaction (PPI) of HK2 vs p62 and HMGCS1 vs p62 were strong with the free energy Δtotal_HK2_ = −96.54 kcal/mol and Δtotal_HMGCS1_ = −56.87 kcal/mol, respectively (Fig. 4D, E). The HK2-p62 and HMGCS1-p62 complexes have definitely already both reached the state of equilibrium by 300 ns by RMSD analysis, indicating the relative adequacy of the simulation duration(Fig. 4F). RMSF analysis showed that the two complexes have better protein flexibility with similar peak shapes both basing on p62, which depicted the dynamic binding of protein complexes (Fig. 4G). During the molecular simulation process (within the 300 ns), there was a significant change in the number of hydrogen bonds, showing an obvious change in the geometric structure of the protein itself, which also reflected the stability of PPI (Fig. 4H). There were also many protein residues contributing to the PPI. The greatest one was arginine(769) [ARG(769)] in HK2-p62 complex and ARG(313) in HMGCS1-p62 complex, respectively (Fig. 4I, J). We visualize the entire MDS process of the two complexes (Fig. 4K). These data showed that HMGCS1 and HK2 may have mutual influences on each other, but this influence is not for the same domain on p62, but rather due to spatial hindrance caused by proximity of the binding regions, leading to changes in protein binding ability.

**Fig. 5:**
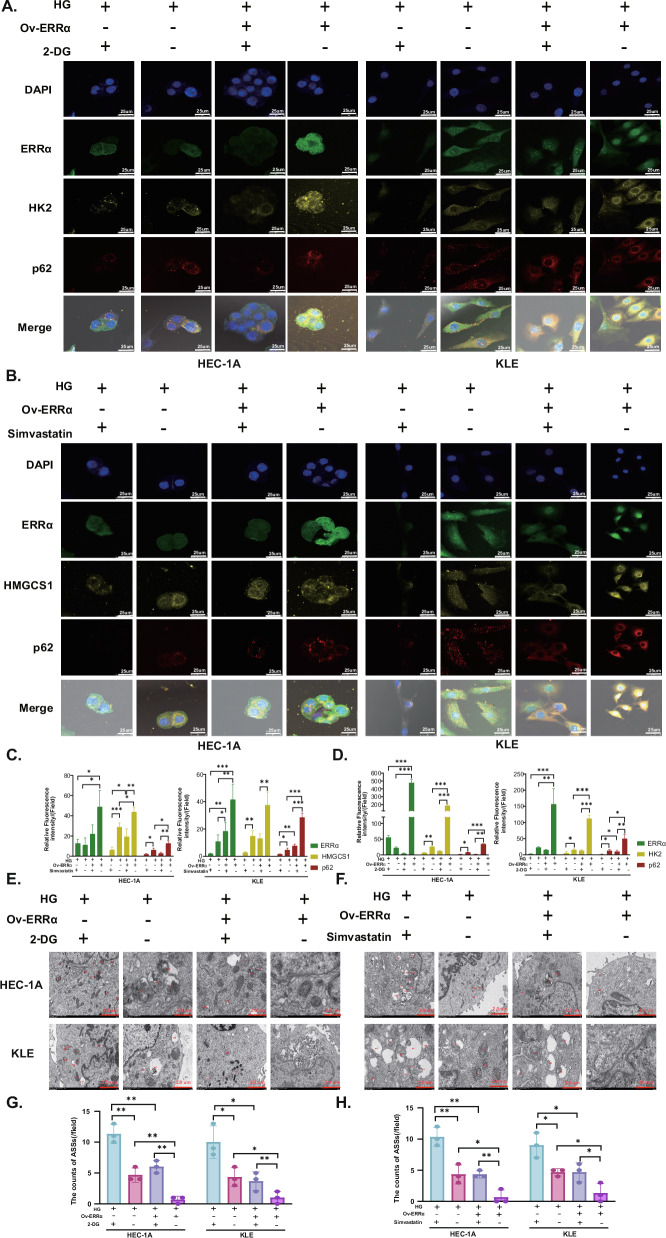
ERRα regulates glucose and cholesterol metabolism to suppress the autophagy–lysosomal pathway. **A** In the HEC-1A and KLE cells, the expression of HK2, p62 treated with 2-DG after ERRα overexpressing in the HG environment was shown by immunofluorescence (Magnification: 80×). **B** In the HEC-1A and KLE cells, the expression HMGCS1, p62 treated with simvastatin was presented by immunofluorescence staining after ERRα up-regulating in the HG environment (Magnification: 80×). **C** Relative fluorescence intensity of ERRα, HK2, and p62 when treated with 10 mM 2-DG for 24 h under high glucose in HEC-1A and KLE cells. **D** Relative fluorescence intensity of ERRα, HMGCS1, and p62 when treated with 5 μM and 10 μM simvastatin for 24 h under high glucose in HEC-1A and KLE cells, respectively. **E**, **F** TEM images of ASSs treated with 2-DG or simvastatin for 24 h under high glucose in KLE and HEC-1A cells, respectively (Magnification: 8000×). **G**, **H** The ASSs counts of HEC-1A and KLE cells treated with 2-DG or simvastatin for 24 h under high glucose after ERRα overexpressed, respectively. *n* = three independent experiments, *P < 0.05, **P < 0.01 or ***P < 0.001. EC endometrial cancer, Mnt mannitol, NG normal glucose, HG high glucose, TEM transmission electron microscopy, 2-DG 2-deoxy-d-arabino-hexose.

After administration of the glycolysis inhibitor 2-deoxy-D-glucose (2-DG), the expression of HK2 and p62 was inhibited in the HG environment, and this effect was attenuated when ERRα was overexpressed. Similarly, when the cholesterol synthesis pathway inhibitor simvastatin was used to inhibit the expression of HMGCS1, the p62 fluorescence intensity decreased (Fig. 5A–D). Second, by TEM, we found that the number of ASSs increased when HK2 and HMGCS1 were inhibited in the HG environment (Fig. 5E–H). The above data suggest that glucose and cholesterol metabolism is regulated by the promotion of ERRα expression in HG environments and that in such an environment, both HK2 and HMGCS1 can bind to p62 and reduce their entry into ASSs to prevent their degradation.

### Targeting of ERRα combined with carboplatin to treat EC organoids

**Fig. 6:**
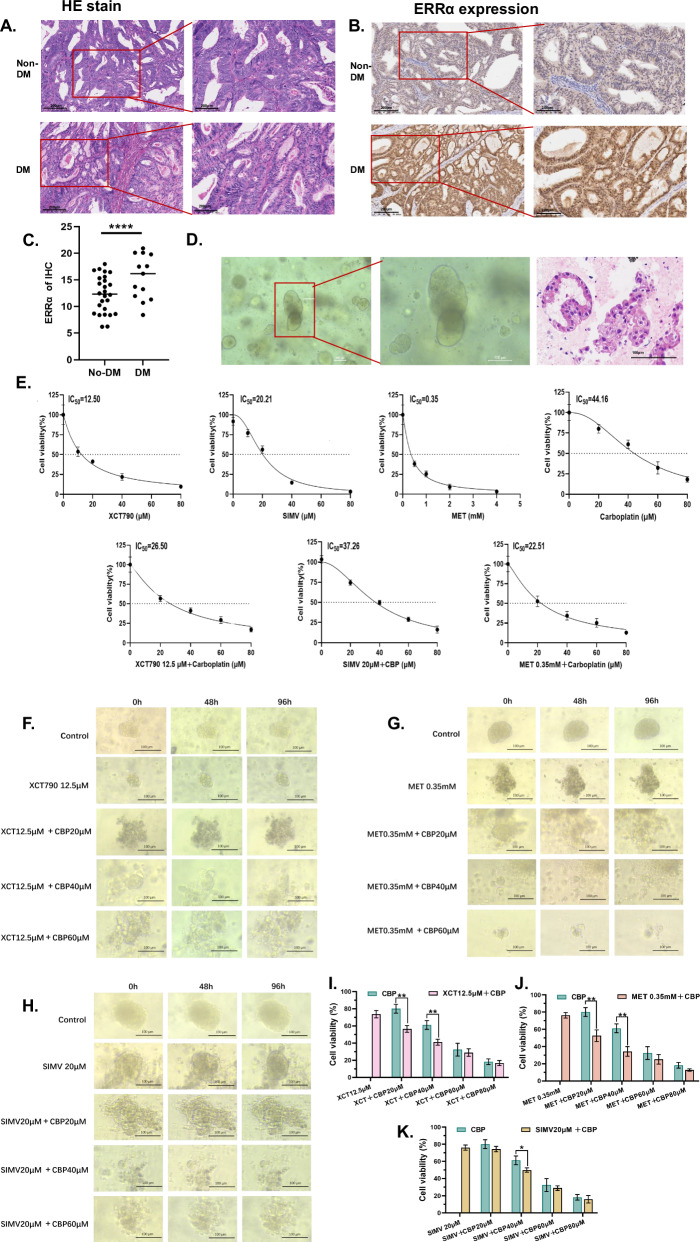
ERRα inhibitor treatment ameliorates the effects on EC with DM-like organoids. **A** H&E staining of tumor sections from EC patients without and with DM. **B** Representative immunohistochemical images of ERRα in EC patients without and with DM. **C** IRS of ERRα was calculated and compared between the patients with and without DM. **D** The cellular morphology of organoids at different magnifications was evaluated using a light microscope and H&E staining. **E** After different treatments, the IC_50_ values in the corresponding groups of organoids were determined using a CCK8 assay. **F**–**H** The morphological changes in EC organoids at 0 h, 48 h, and 96 h were evaluated using light microscopy after treatment with XCT790 (12.5 µM) alone as well as in combination with a carboplatin concentration gradient, metformin (0.35 mM) alone as well as in combination with a carboplatin concentration gradient and simvastatin (20 µM) alone as well as in combination with a carboplatin concentration gradient, respectively. **I**–**K** The cell viability treated with different treatments was evaluated by CCK8 with three replications. The data are shown as the means ± standard deviations. Statistical test: paired *t* test. *p < 0.05, **p < 0.01, ***p < 0.001, ****p < 0.0001. EC endometrial cancer, H&E hematoxylin and eosin, IRS Immunoreactivity score.

We applied the principle of random sampling and performed a 1:1 case‒control matched analysis of 30 patients with DM who had complete pathology specimens available. The patients with DM were accompanied by a higher TC and deeper MI (Table 3). The level of ERRα expressed in tissues was higher in EC patients with DM than those in EC patients without DM (*p* < 0.05, Fig. 6A–C). In clinical practice, the main chemotherapeutic agent used to treat EC patients is carboplatin (CBP) [31, 32]. However, carboplatin resistance, which leads to decreased treatment efficacy, is a frequently emerging problem in the treatment of EC patients. Further, there are differences in treatment response [33]. To explore the relationship between ERRα expression and the response to CBP therapy, we simulated the in vivo microenvironment in EC patients with DM generating a three-dimensional organoid model. The successful generation of organoids was confirmed by light microscopy and HE staining (Fig. 6D) The IC_50_ values of XCT790 (an inhibitor of ERRα), simvastatin (SIMV), metformin (MET), and carboplatin in the EC-like organoids were 12.50 µM, 20.21 µM, 0.35 mM and 44.16 µM, respectively. Then we treated the organoids with XCT790, SIMV, or MET at their IC_50_, and respectively combined them with cisplatin to identify that the IC_50_ of cisplatin ranged from 20 to 40 µM. (Fig. 6E). Then, the organoids were then divided into 3 groups for culture under HG (25 mM glucose) conditions. The three groups of EC-like organoids were treated with XCT790, MET, or SIMV combined with different doses of carboplatin, and the growth of the organoids was recorded at 48 h and 96 h. In terms of morphology, a significant disintegration of the three-dimensional (3D) structure of the organoids was observed with time and increasing dose of carboplatin, accompanied by an obvious decrease in the organoid volume in XCT790 + carboplatin groups (Fig. 6F). Similarly, there was also an obvious disintegration of the organoids’ 3D structure in MET + carboplatin groups. The volume of the organoids decreases with a dose and time-dependent manner with more debris (Fig. 6G). However, as time and dose increased, the organoids could maintain their 3D structure in the SIMV+carboplatin groups except SIMV + 40 μM carboplatin group, which had a nearly full disintegration of organoids. (Fig. 6H). In addition, the cell viability of the organoids with carboplatin combination therapy decreased significantly in a dose-dependent manner. Consistently, the effective dose of cisplatin in the combination strategy were 20 and 40 µM in both XCT790 and MET groups, while the therapeutic dose of cisplatin in the SIMV group was 40 µM that was a higher dose with higher cytotoxicity (Fig. 6I–K). Though the cell viability of XCT790 + 20 µM carboplatin treatment was similar to that of MET + 20 µM carboplatin, the bigger volume of organoids was more visible in the MET + carboplatin group, which indicated the XCT790 + 20 µM carboplatin had the better inhibition of organoids.

**Table 3:** Correlation between diabetes mellitus and the clinicopathologic characteristics of endometrial cancer patients.

Characteristics	Non DM (*n* = 30)	DM (*n* = 30)	*p* Value
*Cholesterol abnormalities, n (%)*			**0.037**
<5.2 (mmol/L)	21 (70%)	13 (43%)	
≥5.2 (mmol/L)	9 (30%)	17 (57%)	
*Triglyceride abnormalities, n (%)*			0.121
<1.7 (mmol/L)	19 (63%)	13 (43%)	
≥1.7 (mmol/L)	11 (37%)	17 (57%)	
Age at diagnosis, median (IQR)	56 ± 6	59 ± 7	0.087
*Menopausal status, n (%)*			0.080
No	11 (37%)	5 (17%)	
Yes	19 (63%)	25 (83%)	
BMI, median (IQR)	24.9 ± 3.9	25.7 ± 2.4	0.321
*FIGO stage, n (%)*			0.195
I–II	25 (83%)	29 (97%)	
III–IV	5 (17%)	1 (3%)	
*Grade, n (%)*			0.486
G1	11 (48%)	9 (33%)	
G2	9 (39%)	15 (56%)	
G3	3 (13%)	3 (11%)	
*CSI, n (%)*			0.095
No	22 (73%)	27 (90%)	
Yes	8 (27%)	3 (10%)	
*MI, n (%)*			**0.028**
No	27 (90%)	20 (67%)	
Yes	3 (10%)	10 (33%)	
*LVSI, n (%)*			0.424
No	25 (83%)	28 (93%)	
Yes	5 (17%)	2 (7%)	
*LNM, n (%)*			0.612
No	27 (90%)	29 (97%)	
Yes	3 (10%)	1 (3%)	

*DM* diabetes mellitus, *BMI* body mass index, *FIGO* international Federation of Gynecology and obstetrics, *CSI* cervical stromal involvement, *MI* myometrial infiltration, *LVSI* lymphovascular interstitial infiltration, *LNM* lymph node metastasis, *p* < 0.05 is considered significantly different with bold value.

## Discussion

In the present study, we found that the HG environment in EC is directly connected to increased tumoral ERRα expression and suppressed ASS pathway. Analyzing clinical data, we further showed that in patients with constant high blood glucose levels like DM, ERRα expression promotes MI in EC, thereby representing a poor prognostic factor [34]. Research has discussed disrupted dynamin 1 expression leading to mitochondrial disorganization and promoting the alteration of cellular metabolism, including the epithelial–mesenchymal transition (EMT) process in EC cells as causative for MI [35]. HG environment increases the expression of GLUT4 and estrogen receptors (ER) in EC cells, which can affect the endocrine function of the EC tissue [36]. Of note, ERRα expression is also strongly associated with other estrogen-dependent tumors, such as breast cancer [19, 37].

Here we describe ERRα as a novel key player in the context of glucose and cholesterol metabolism in EC. We found that HG stimulation induces increased ERRα expression levels and promotes glucose and cholesterol metabolism. This is supported by our previous work that demonstrated that the TFEB/ERRα axis alters lipid metabolism in EC cell membranes [38]. The ERRα/HMGCS1 axis regulates cholesterol metabolism, promoting EC invasion and metastasis [20]. In addition, high expression of ERRα promotes glycolytic processes while affecting the pyroptosis signaling pathway, leading to drug resistance in EC cells [39].

A HG microenvironment forwards EC cell migration and invasion via energy metabolism pathways [40, 41]. In line with our findings, studies in renal cell carcinoma have shown that glycolysis is converted to cholesterol in an acidic environment [42], and the overall survival time was shorter in the high TC group [43]. However, changes in glucose metabolism are more disruptive than changes in cholesterol metabolism [44, 45]. HK2, which is an enzyme in glycolysis, is not degraded in the presence of defective autophagy and thus promotes lactate accumulation and accelerates hepatocarcinogenesis by binding to p62 [46]. In this study, we found that HK2 and HMGCS1 inhibited the elimination of ASSs by binding to P62 to prevent reductions in the lactate and cholesterol concentrations, respectively, and nutrient supplementation provided conditions sufficient for the growth of cancer cells. In addition, we provide evidence for a specific mechanism explaining the relationship between HMGCS1 and autophagy by demonstrating that HMGCS1 negatively regulates autophagy in an HG environment.

Autophagy, a process involved in the intracellular removal of useless proteins and organelles, either promotes or inhibits tumorigenesis [47]. The autophagy receptor protein p62 is a degradation product of late autophagy, and its expression is negatively correlated with autophagic activity [48]. We showed that autophagy in EC cells was inhibited in an HG environment. Under these conditions, higher levels of ERRα expression are associated with lower levels of autophagy. It has also been suggested that ERRα participates in the activation of autophagy during mycobacterial growth [49]. However, in that study, autophagy was protecting normal bodily functions. Moreover, inhibition of ERRα expression using XCT790 in the setting of Parkinson’s disease can increase the protective effect of autophagy on the nervous system [50]. This finding supports our assertion that our results will lead to further study of autophagy in EC.

Treatment with Metformin has been demonstrated to be beneficial in EC patients with comorbid DM by causing hypoglycemia and weight loss and exerting antitumor effects [51–53]. However, it has also been shown that the inhibitory effect of metformin on EC cell growth is less significant under HG conditions than under NG conditions [25]. Recently, we discovered that the ERRα inhibitor XCT790 is more effective than metformin for the treatment of ER-positive EC [19]. Consistent with these previous results, in the present study, in the HG group, the fragmentation of EC organoids after treatment with XCT790 in combination with carboplatin was comparable to that after treatment with metformin combined with carboplatin, indicating that the addition of the ERRα inhibitor XCT790 could be considered for the method of patients with metabolic syndrome accompanied by EC.

In conclusion, in the present study, we found that EC patients with DM have disorders of glucose and cholesterol metabolism that are regulated by ERRα. In this population, the expression of ERRα is increased, which promotes increases in glucose and cholesterol metabolism. Autophagy is one important pathway for removing excess metabolites, but the autophagy‒lysosomal pathway is inhibited; subsequently, the intracellular lactate and cholesterol concentrations increase, which provides an abundant supply of nutrients for EC cells. Combined with our group’s previous research, these findings indicate that ERRα might serve as a potential therapeutic target in EC independent of comorbid DM. These findings will help to identify new therapeutic combinations for future clinical diagnosis and treatment. However, this study also has limitations. First, this was a single-center cohort investigation. Second, the mechanism by which an HG microenvironment results in increased ERRα expression has not been fully elucidated. Finally, the therapeutic effect of the ERRα inhibitor XCT790 combined with carboplatin was assessed only at the organoid level. In this regard, further investigations are needed.

## Supplementary information

Original WB Data

## Data Availability

Informed consent was obtained from all subjects involved in the study. Data availability statement: The data presented in this study are available upon request from the corresponding author. The data are not publicly available due to privacy concerns.
