# Supplementary material for: High glucose levels promote glycolysis and cholesterol synthesis via ERRα and suppress the autophagy–lysosomal pathway in endometrial cancer
Source: Cell Death Dis. 2025 Mar 17;16(1):182. doi: 10.1038/s41419-025-07499-y (PMC11914573; doi:10.1038/s41419-025-07499-y)
Supplement: Supplementary file 1 — Original WB Data [file 41419_2025_7499_MOESM1_ESM.pdf]

Fig2.

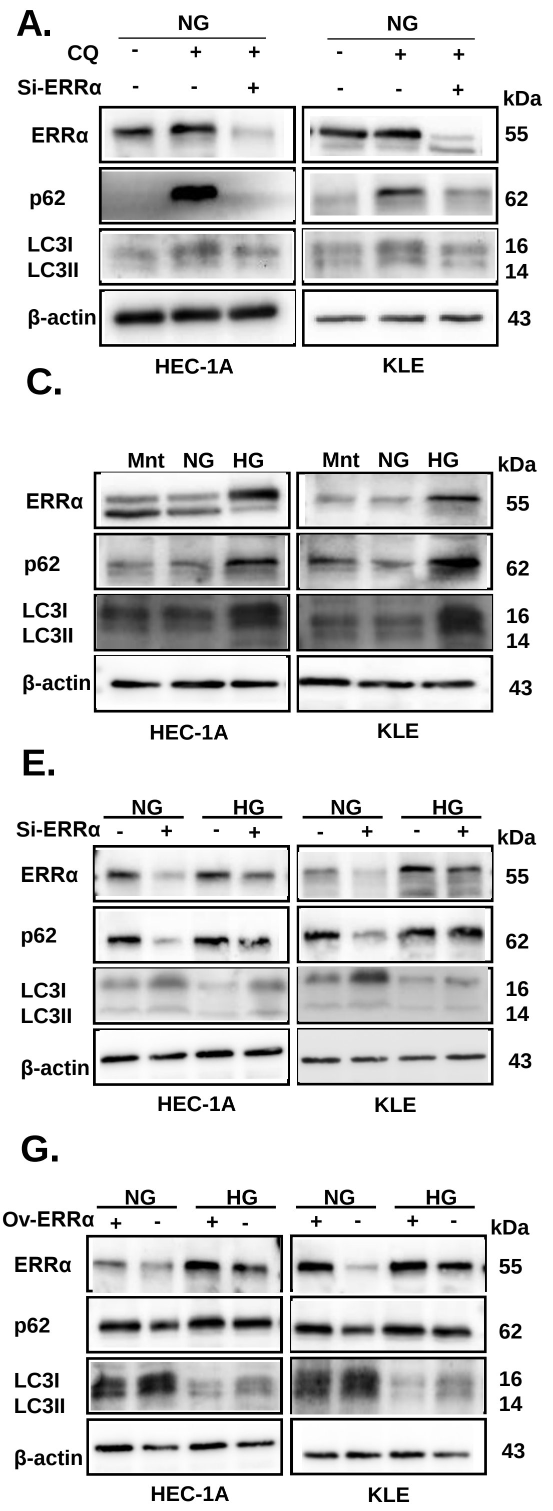
A.
NG
NG
CQ - + + - + +
Si-ERRα - - + - - +
kDa
ERRα 55
p62 62
LC3I 16
LC3II 14
β-actin 43
HEC-1A
KLE
C.
Mnt NG HG
Mnt NG HG
kDa
ERRα 55
p62 62
LC3I 16
LC3II 14
β-actin 43
HEC-1A
KLE
E.
NG HG NG HG
Si-ERRα - + - + - + - +
kDa
ERRα 55
p62 62
LC3I 16
LC3II 14
β-actin 43
HEC-1A
KLE
G.
NG HG NG HG
Ov-ERRα + - + - + - + -
kDa
ERRα 55
p62 62
LC3I 16
LC3II 14
β-actin 43
HEC-1A
KLE

Fig3.

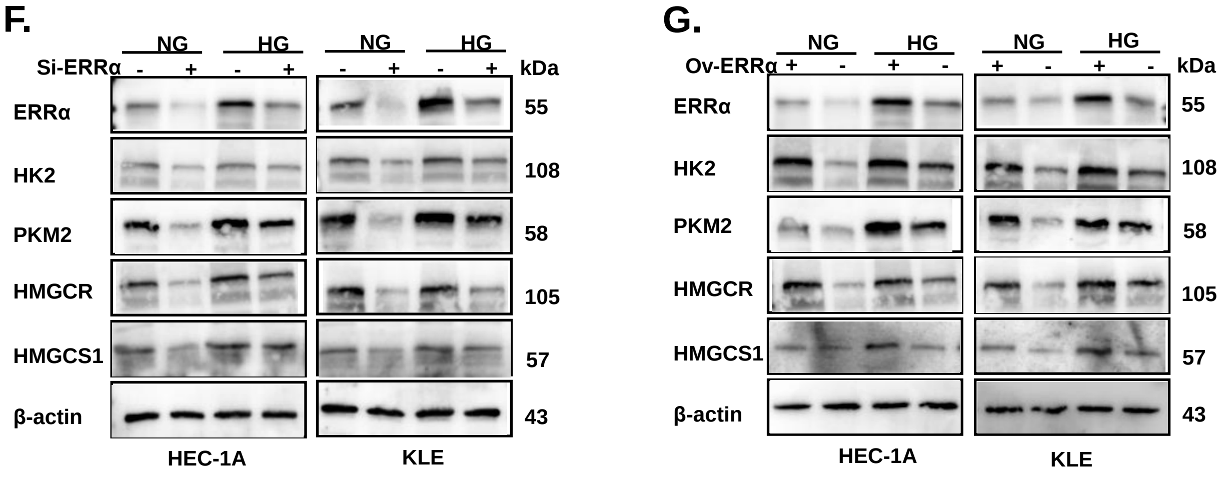
F.
NG HG NG HG
Si-ERRα - + - + - + - +
kDa
ERRα 55
HK2 108
PKM2 58
HMGCR 105
HMGCS1 57
β-actin 43
HEC-1A
KLE
G.
NG HG NG HG
Ov-ERRα + - + - + - + -
kDa
ERRα 55
HK2 108
PKM2 58
HMGCR 105
HMGCS1 57
β-actin 43
HEC-1A
KLE

Fig4.

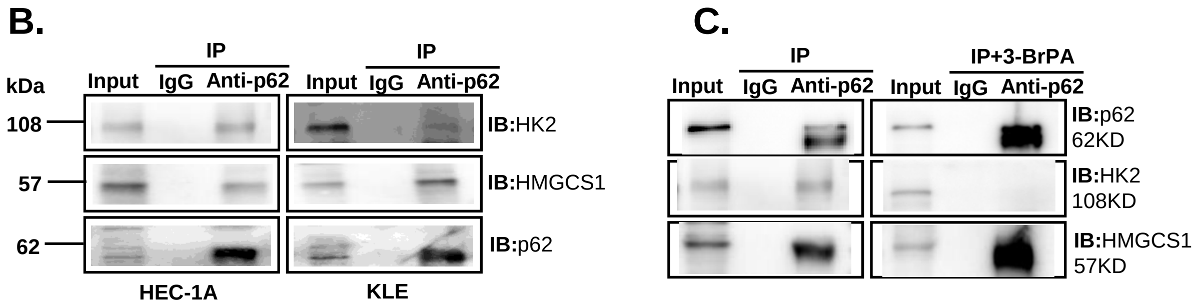
B.
IP
IP
kDa
Input
IgG
Anti-p62
Input
IgG
Anti-p62
108
IB:HK2
57
IB:HMGCS1
62
IB:p62
HEC-1A
KLE
C.
IP
IP+3-BrPA
Input
IgG
Anti-p62
Input
IgG
Anti-p62
IB:p62
62KD
IB:HK2
108KD
IB:HMGCS1
57KD

Fig2A

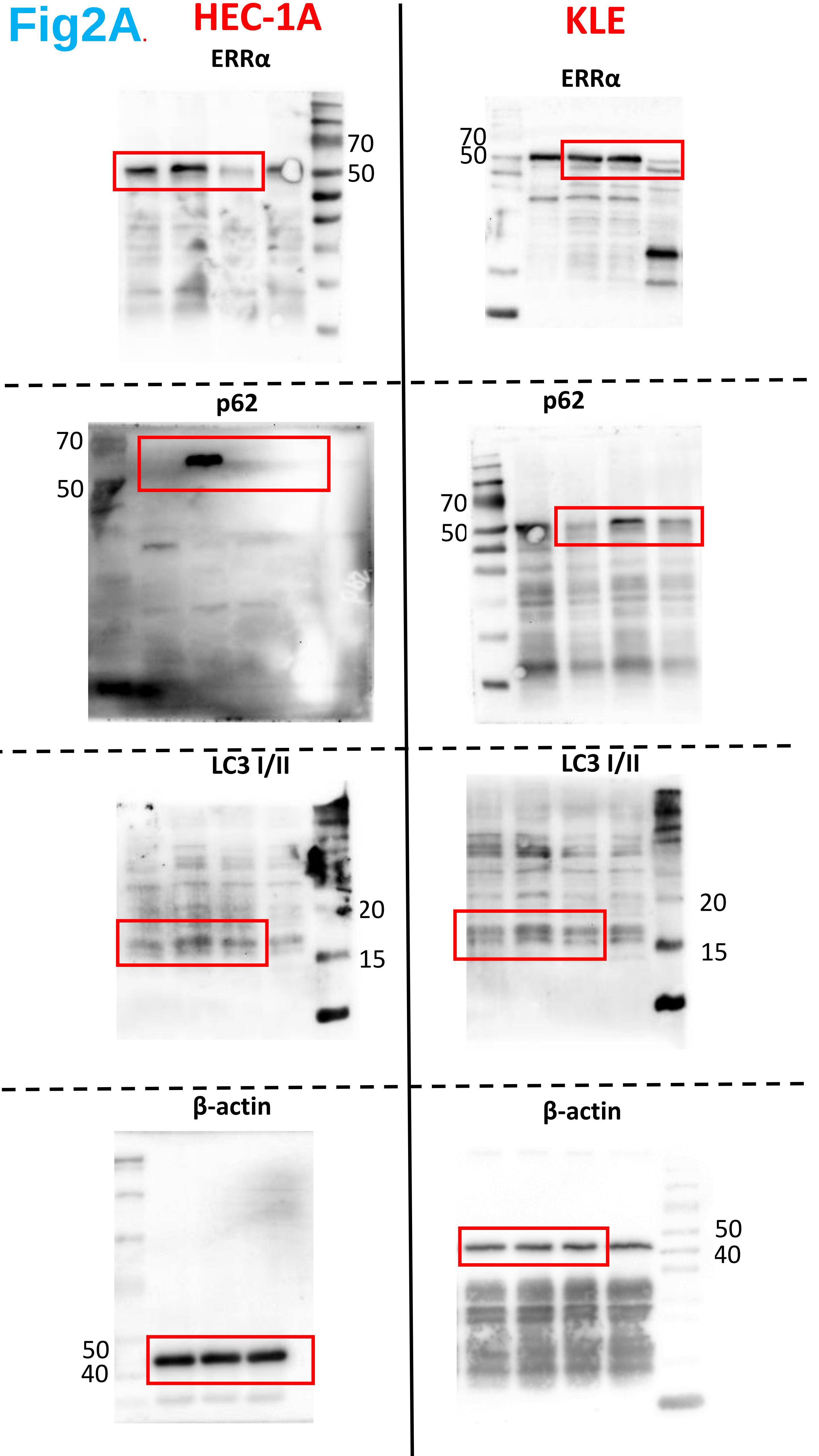
HEC-1A
KLE
ERRα
70
50
ERRα
70
50
p62
70
50
p62
70
50
LC3 I/II
20
15
LC3 I/II
20
15
β-actin
50
40
β-actin
50
40

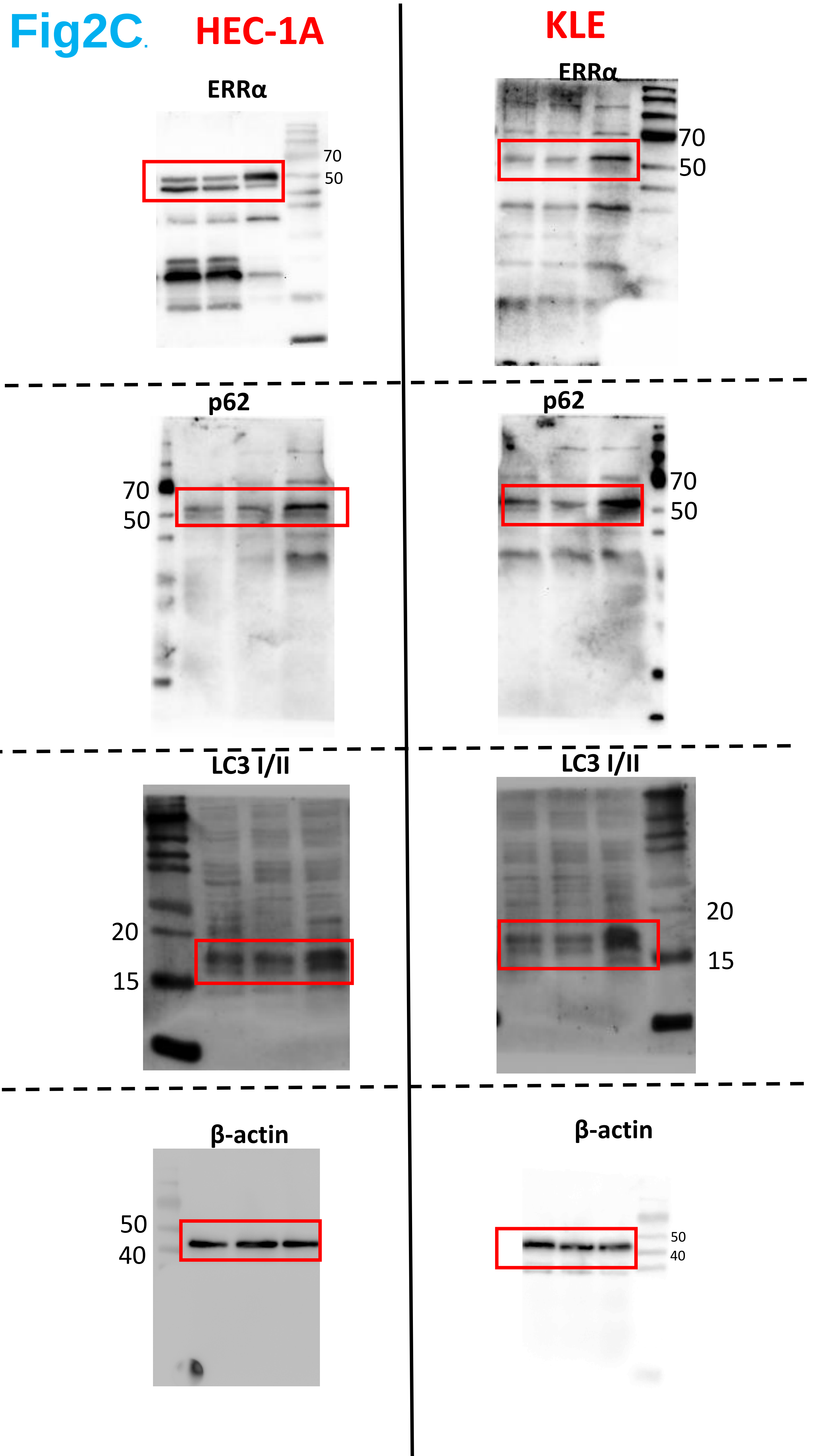

Fig2C.
HEC-1A
KLE
ERRα
70
50
p62
70
50
LC3 I/II
20
15
β-actin
50
40

Fig2E.

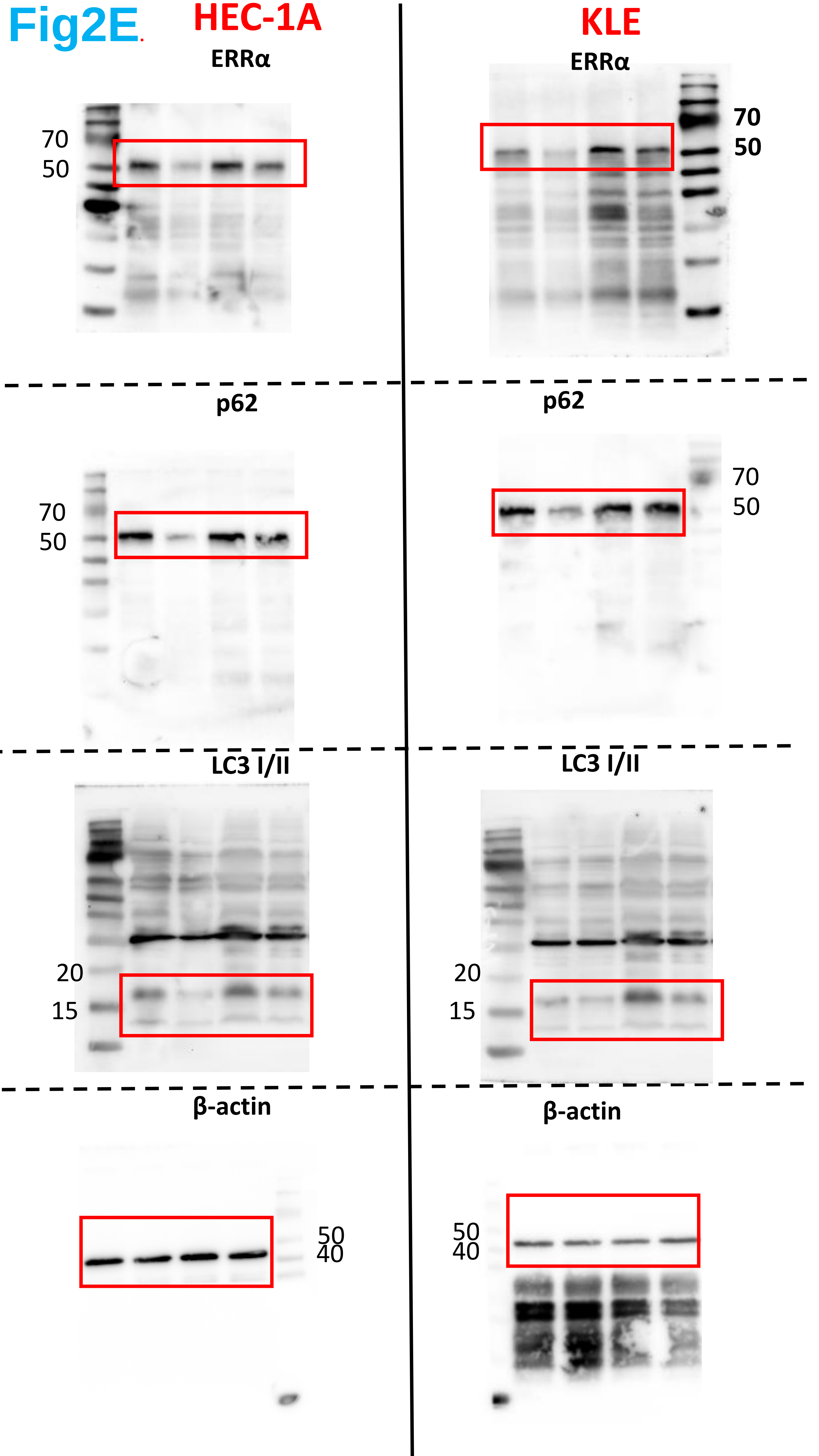
HEC-1A
KLE
ERRα
70
50
ERRα
70
50
p62
70
50
p62
70
50
LC3 I/II
20
15
LC3 I/II
20
15
β-actin
50
40
β-actin
50
40

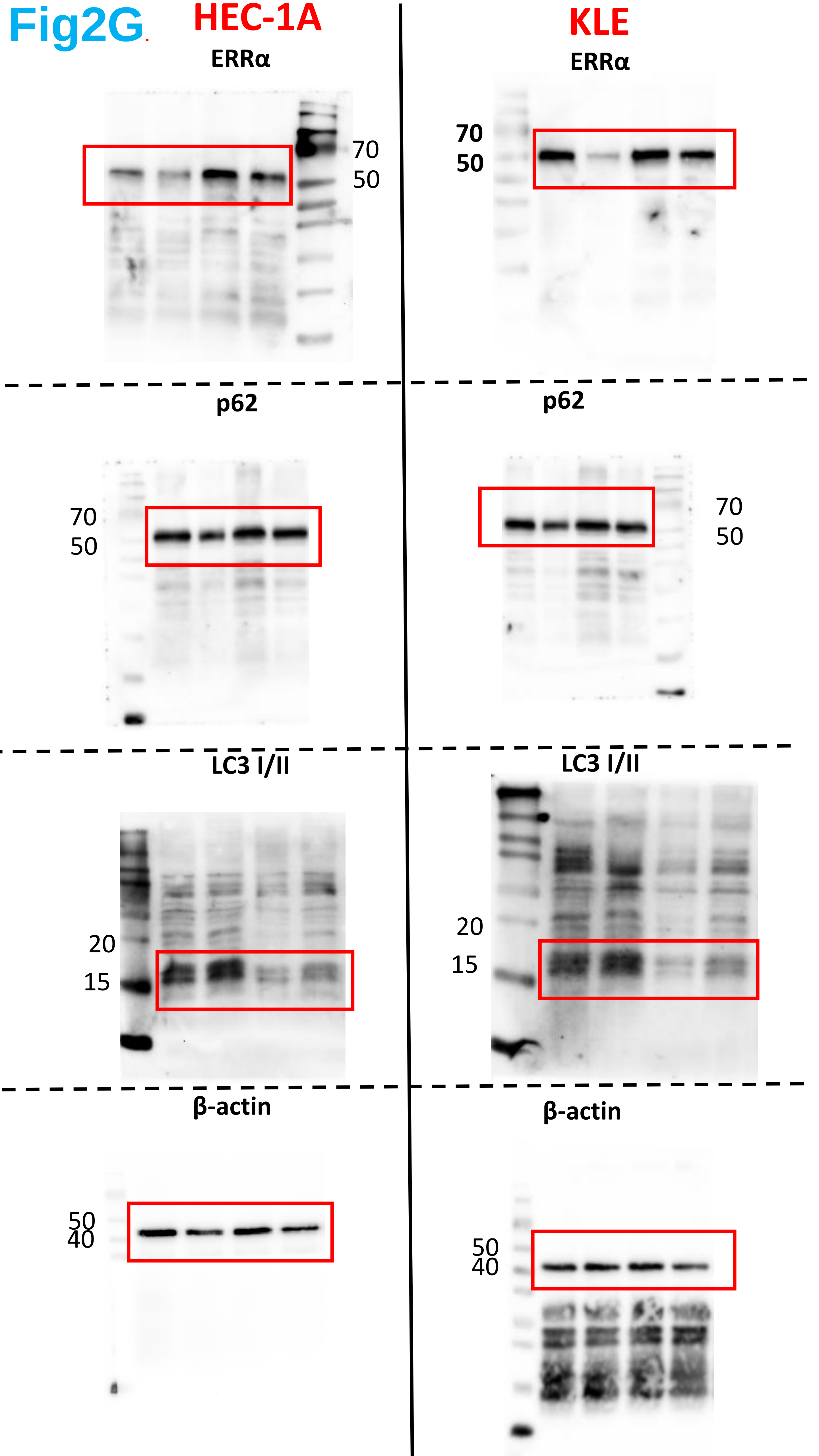
Fig2G.
HEC-1A
KLE
ERRα
ERRα
70
50
70
50
p62
p62
70
50
70
50
LC3 I/II
LC3 I/II
20
15
20
15
β-actin
β-actin
50
40
50
40

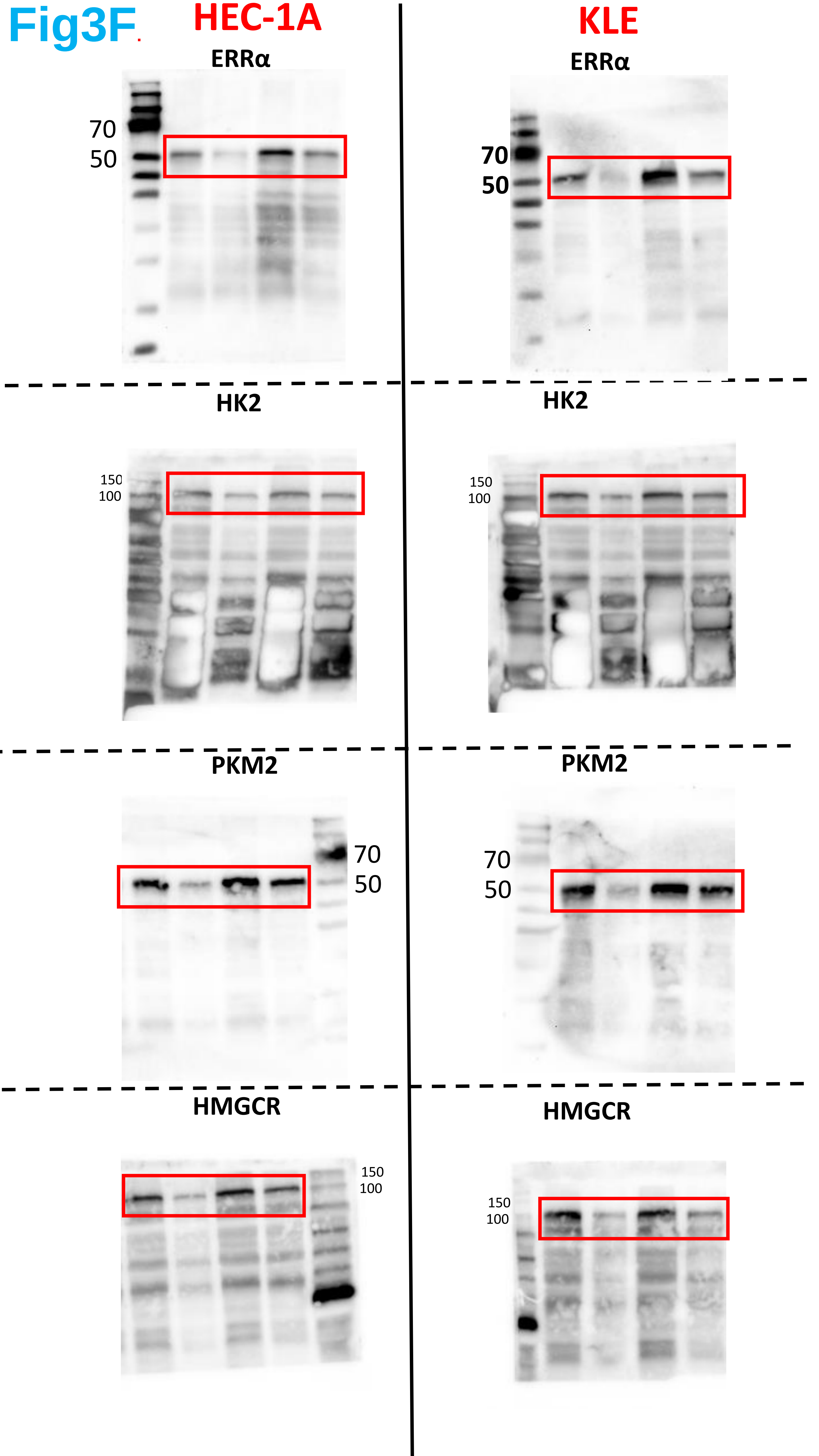
Fig3F
HEC-1A
KLE
ERRα
70
50
ERRα
70
50
HK2
150
100
HK2
150
100
PKM2
70
50
PKM2
70
50
HMGCR
150
100
HMGCR
150
100

Fig3F

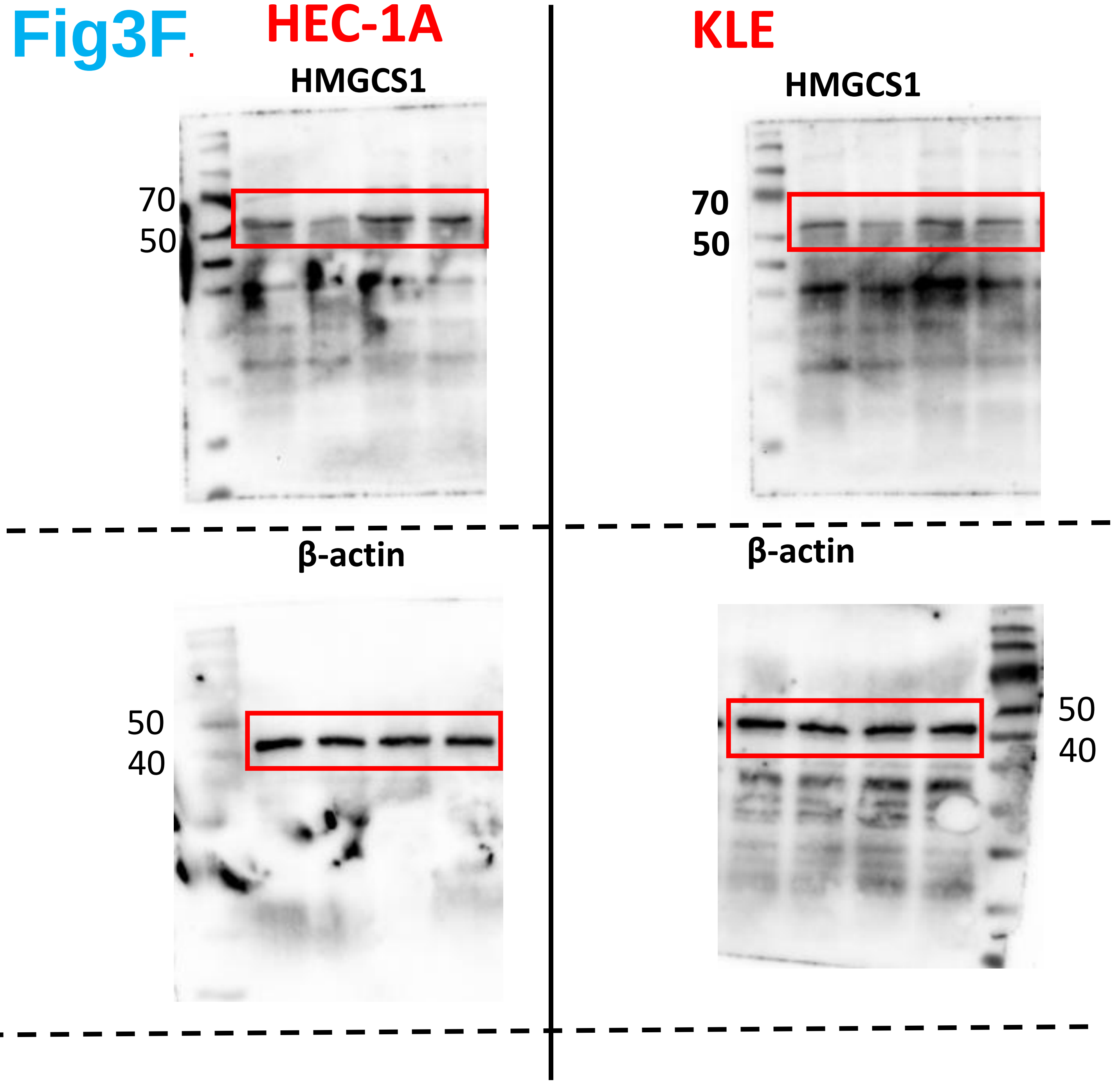

Fig3G

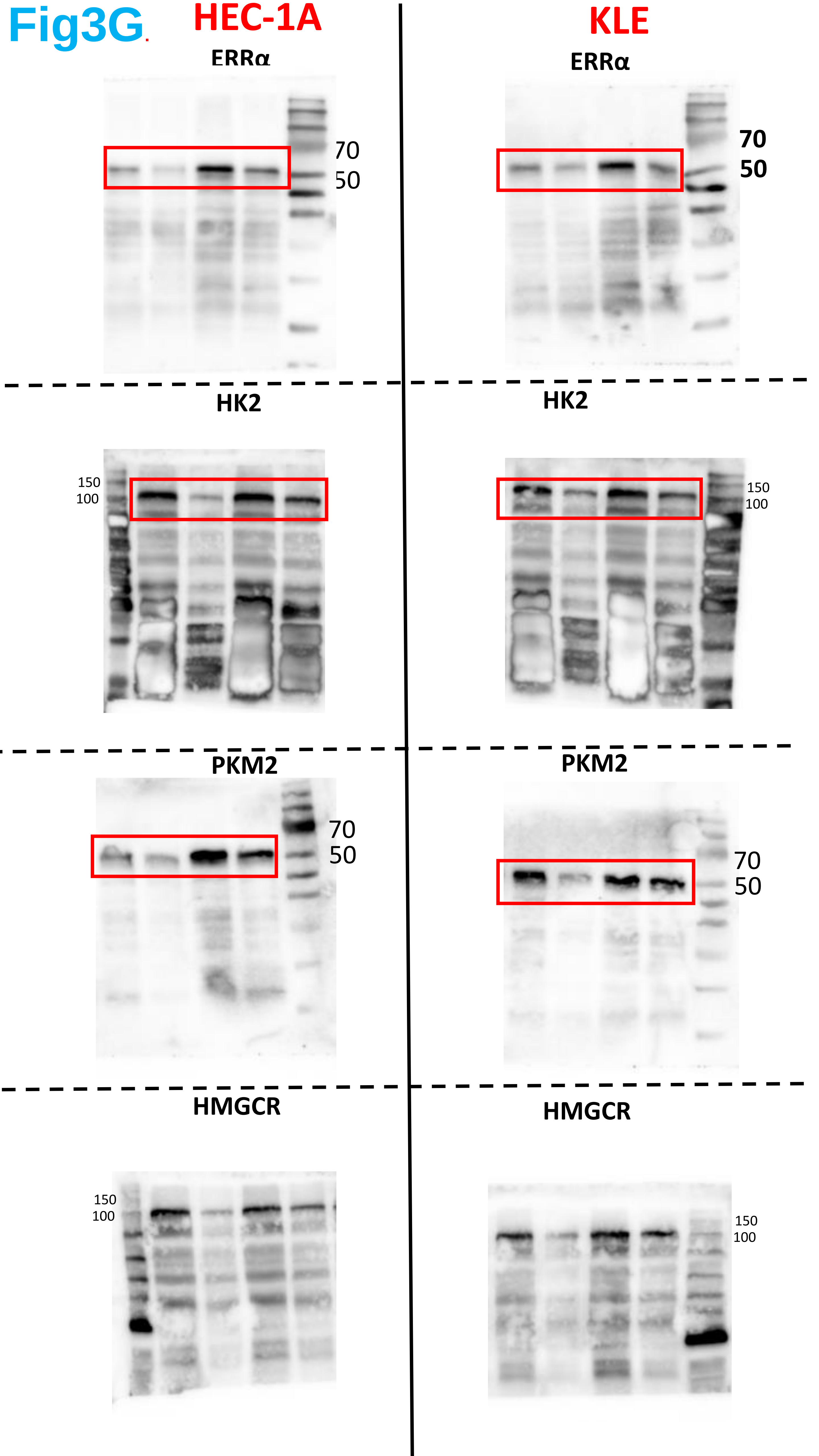
HEC-1A
KLE
ERRα
70
50
ERRα
70
50
HK2
150
100
HK2
150
100
PKM2
70
50
PKM2
70
50
HMGCR
150
100
HMGCR
150
100

Fig3G

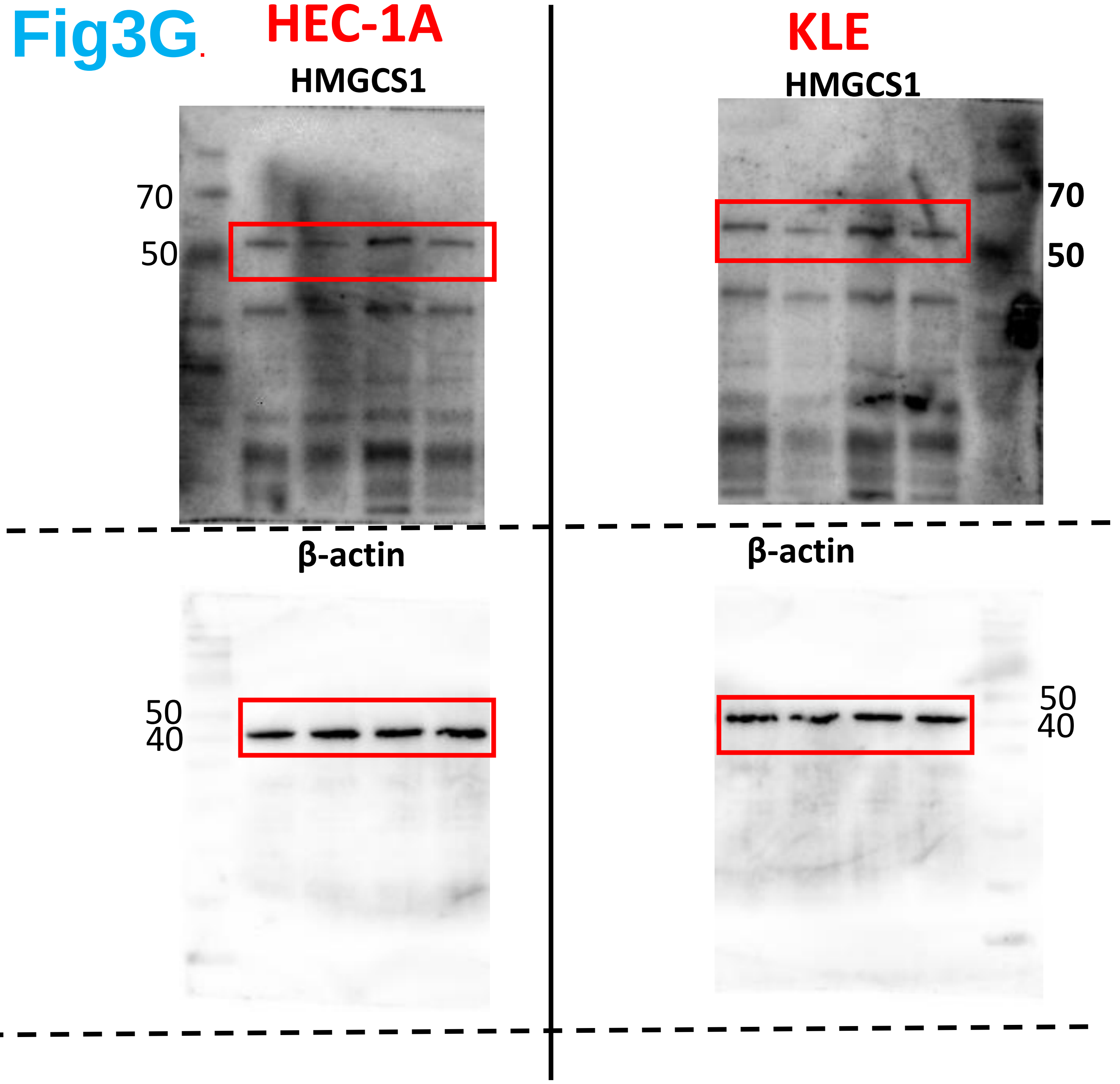

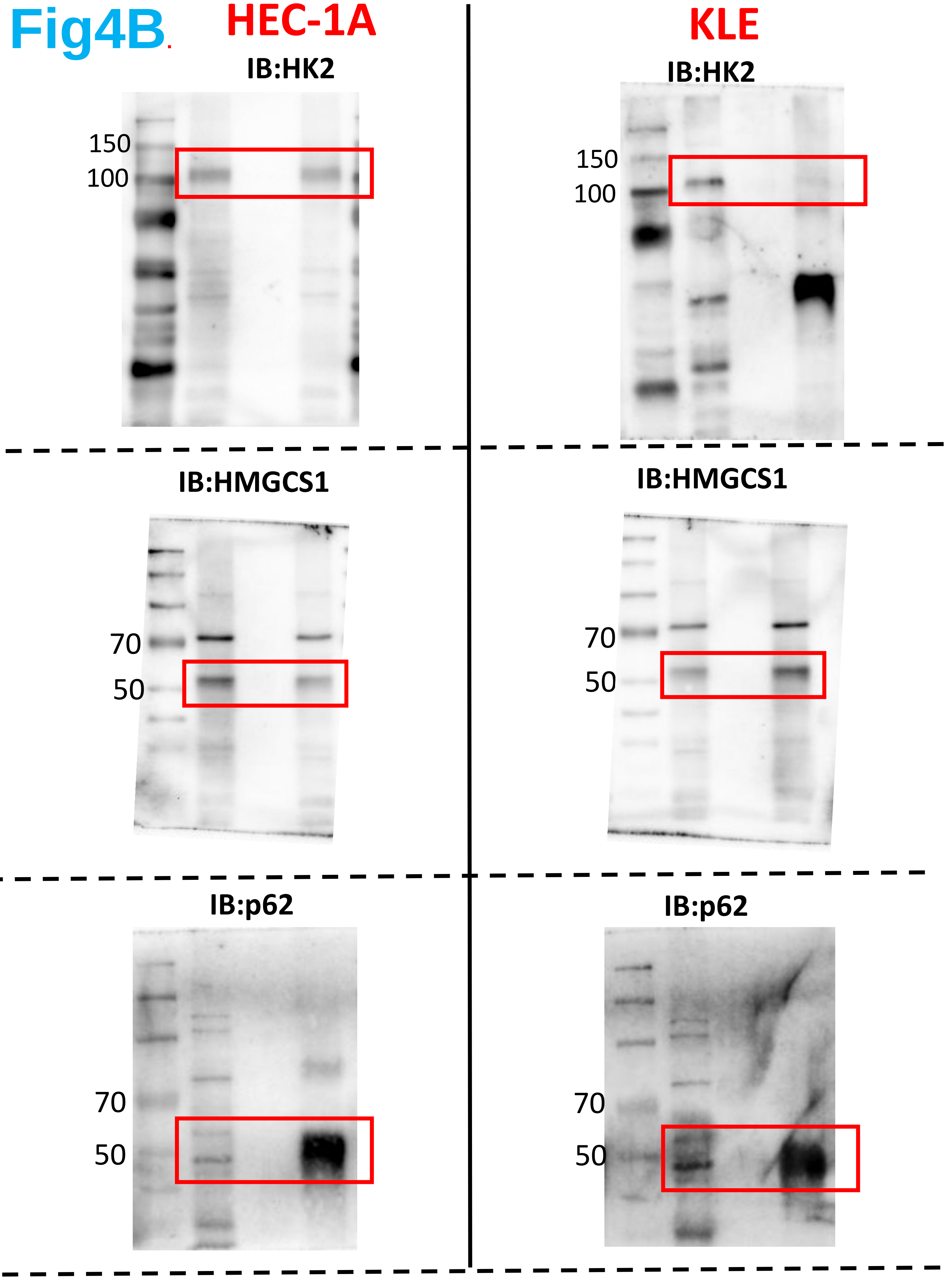
Fig4B.
HEC-1A
KLE
IB:HK2
150
100
IB:HK2
150
100
IB:HMGCS1
70
50
IB:HMGCS1
70
50
IB:p62
70
50
IB:p62
70
50

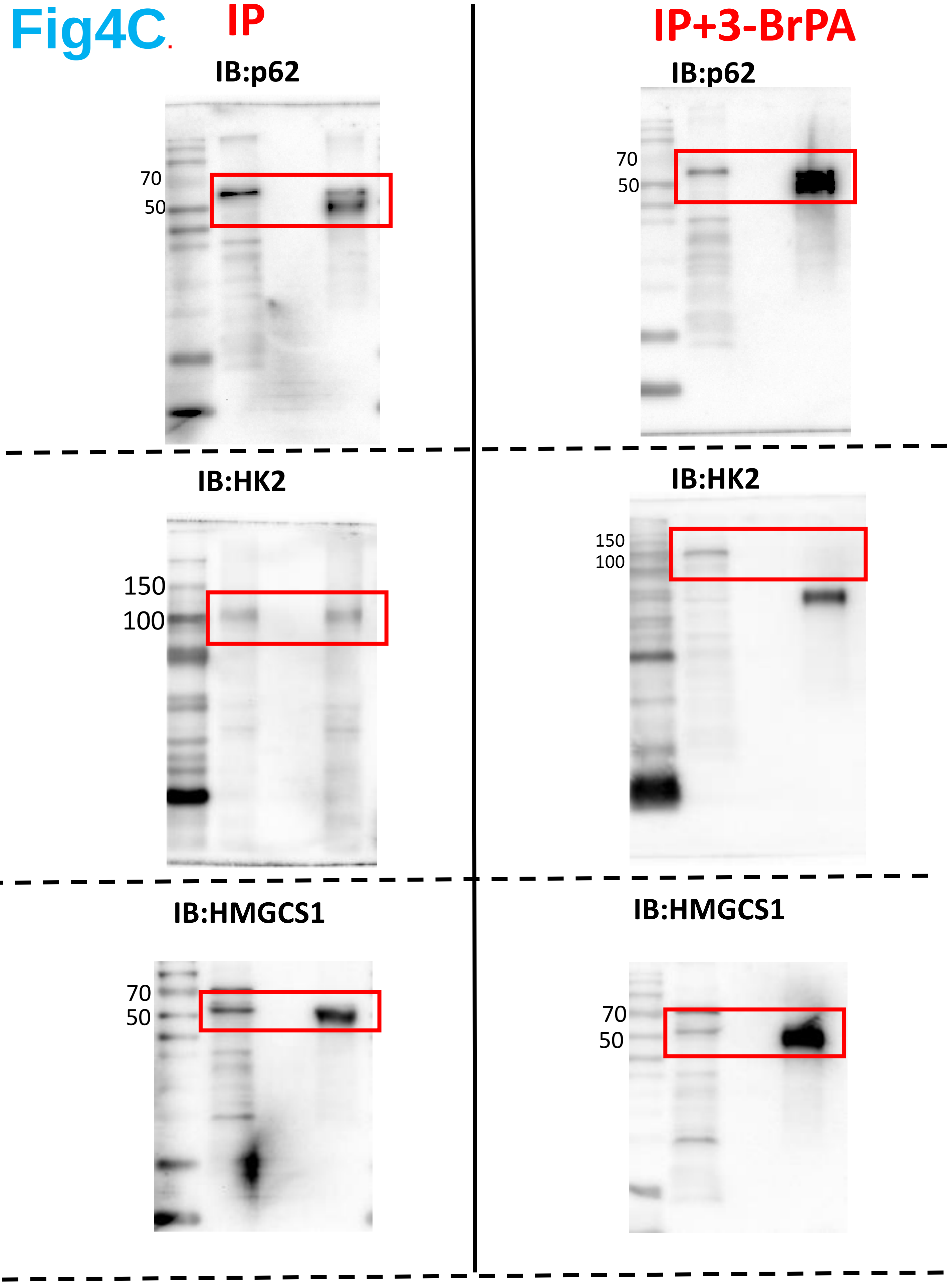
Fig4C
IP
IP+3-BrPA
IB:p62
70
50
IB:p62
70
50
IB:HK2
150
100
IB:HK2
150
100
IB:HMGCS1
70
50
IB:HMGCS1
70
50
